# Parasite Detection in Visceral Leishmaniasis Samples by Dye-Based qPCR Using New Gene Targets of *Leishmania infantum* and *Crithidia*

**DOI:** 10.3390/tropicalmed8080405

**Published:** 2023-08-08

**Authors:** Nayore Tamie Takamiya, Luana Aparecida Rogerio, Caroline Torres, João Augusto Franco Leonel, Geovanna Vioti, Tricia Maria Ferreira de Sousa Oliveira, Karoline Camila Valeriano, Gabriane Nascimento Porcino, Isabel Kinney Ferreira de Miranda Santos, Carlos H. N. Costa, Dorcas Lamounier Costa, Tauana Sousa Ferreira, Rodrigo Gurgel-Gonçalves, João Santana da Silva, Felipe Roberti Teixeira, Roque Pacheco De Almeida, José M. C. Ribeiro, Sandra Regina Maruyama

**Affiliations:** 1 Department of Genetics and Evolution, Center for Biological Sciences and Health, Federal University of São Carlos (UFSCar), São Carlos 13565-905, SP, Brazil; nayoretakamiya@estudante.ufscar.br (N.T.T.); frt@ufscar.br (F.R.T.); 2 Post-Graduate Program in Experimental Epidemiology Applied to Zoonoses at the Faculty of Veterinary Medicine and Animal Science, University of São Paulo, São Paulo 05508-270, SP, Brazil; 3 Department of Veterinary Medicine, Faculty of Animal Science and Food Engineering, University of São Paulo, Pirassununga 13635-900, SP, Brazil; 4 Ribeirão Preto Medical School, University of São Paulo, FMRP-USP, Ribeirão Preto 14049-900, SP, Brazil; 5 Natan Portela Institute of Tropical Diseases, Teresina 64002-510, PI, Brazil; 6 Laboratory of Medical Parasitology and Vector Biology, Faculty of Medicine, University of Brasília, Brasília 70910-900, DF, Brazil; 7 Fiocruz-Bi-Institutional Translational Medicine Project, Oswaldo Cruz Foundation, Ribeirão Preto 14040-900, SP, Brazil; 8 Department of Medicine, Center for Biology and Health Sciences, Federal University of Sergipe (UFS), Aracaju 49060-108, SE, Brazil; 9 National Institute of Allergy and Infectious Diseases, National Institutes of Health, NIH/NIAID, Rockville, MD 20892, USA

**Keywords:** visceral leishmaniasis, molecular diagnosis, quantitative PCR (qPCR), *Crithidia* sp. LVH60A, *Leishmania infantum*, trypanosomatid co-infection

## Abstract

Visceral leishmaniasis (VL) is a neglected disease considered a serious public health problem, especially in endemic countries. Several studies have discovered monoxenous trypanosomatids (*Leptomonas* and *Crithidia*) in patients with VL. In different situations of leishmaniasis, investigations have examined cases of co-infection between *Leishmania* spp. and *Crithidia* spp. These coinfections have been observed in a wide range of vertebrate hosts, indicating that they are not rare. Diagnostic techniques require improvements and more robust tools to accurately detect the causative agent of VL. This study aimed to develop a real-time quantitative dye-based PCR (qPCR) assay capable of distinguishing *Leishmania infantum* from *Crithidia*-related species and to estimate the parasite load in samples of VL from humans and animals. The primer LinJ31_2420 targets an exclusive phosphatase of *L. infantum*; the primer Catalase_LVH60-12060_1F targets the catalase gene of *Crithidia*. Therefore, primers were designed to detect *L. infantum* and *Crithidia* sp. LVH60A (a novel trypanosomatid isolated from VL patients in Brazil), in samples related to VL. These primers were considered species-specific, based on sequence analysis using genome data retrieved from the TriTryp database and the genome assembling of *Crithidia* sp. LVH60A strain, in addition to experimental and clinical data presented herein. This novel qPCR assay was highly accurate in identifying and quantifying *L. infantum* and *Crithidia* sp. LVH60A in samples obtained experimentally (in vitro and in vivo) or collected from hosts (humans, dogs, cats, and vectors). Importantly, the screening of 62 cultured isolates from VL patients using these primers surprisingly revealed that 51 parasite cultures were PCR+ for *Crithidia* sp. In addition, qPCR assays identified the co-infection of *L. infantum* with *Crithidia* sp. LVH60A in two new VL cases in Brazil, confirming the suspicion of co-infection in a previously reported case of fatal VL. We believe that the species-specific genes targeted in this study can be helpful for the molecular diagnosis of VL, as well as for elucidating suspected co-infections with monoxenous-like trypanosomatids, which is a neglected fact of a neglected disease.

## 1. Introduction

Leishmaniasis is a neglected disease and is considered an important public health problem caused by the protozoan *Leishmania*. More than 20 species of this genus are known to infect humans and other mammals, and are transmitted to vertebrate hosts by infected phlebotomine sandflies [1,2,3]. The most severe form of the disease is visceral leishmaniasis (VL) due to systemic infection that affects internal organs, such as the spleen, bone marrow, liver, and lymph nodes. It may be fatal when not properly treated [4]. Thirteen countries have been recorded as endemic for VL, including several in East Africa, South Asia, and America, including Brazil, which accounts for 93% of the cases [5]. In Brazil, the etiological agent for VL is *Leishmania infantum* (synonym *L. chagasi*) [1]. VL symptomatology ranges from asymptomatic to mild or oligosymptomatic, up to severe outcomes [5]. Infection by *L. infantum* triggers immunopathological processes that have been elucidated by large-scale gene expression studies [6,7]. Acute infection in symptomatic VL patients has elicited pathways related to interferons (IFN-γ and IFN-β) and neutrophil responses, but interestingly, even asymptomatic individuals with an undetected parasite load have presented considerable molecular disturbance in the gene expression pattern of leukocytes. In addition, long non-coding RNAs (lncRNAs) may act as regulators of immune responses [7].

For the treatment of VL, chemotherapy is administered using liposomal amphotericin B, pentavalent antimonial, and amphotericin B deoxycholate. Another factor to consider is the development of drug resistance by the parasite, mainly because of the failure to accurately identify the etiological agent at the time of diagnosis [8,9,10]. Early diagnosis is of fundamental importance to direct the most appropriate treatment, avoid worsening and progression of the clinical condition, and consequently, reduce mortality in patients with VL [9]. The development of molecular tools has provided complementary and alternative techniques for the diagnosis of VL in research laboratories, particularly in cases of active infection or when conventional diagnoses (parasitological, myelogram, and serological) yield inconclusive results, such as in asymptomatic patients [9,11]. Molecular techniques are reliable due to their high sensitivity and specificity. Among them, we highlight those based on nucleic acid amplification, such as PCR (and its variations) and loop-mediated isothermal amplification (LAMP). Studies have shown that the LAMP assay is promising for the detection of *Leishmania* parasites in tissues and blood [12,13,14]. However, PCR and real-time quantitative polymerase chain reaction (qPCR) are the most commonly used methods for parasite detection. qPCR can detect and measure parasitic load during treatment, assess possible therapeutic failures, and monitor relapse cases. It can also support the screening of asymptomatic individuals because maintenance of the disease involves the circulation of anthropozoonotic reservoirs (domestic animals and humans). Therefore, this molecular tool is essential for both the diagnosis and furthering the understanding of epidemiology in endemic regions [15,16,17].

Cases of co-infection between *Leishmania* spp. and *Crithidia* spp. have been recorded in several vertebrate hosts, including rodents, as reported by Kalantari et al. [18], and humans, as reviewed by Boucinha et al. [19]. The presence of *Crithidia* parasites in patients with cutaneous leishmaniasis from different parts of Iran showed the presence of co-infections (observed by the isolation of *Crithidia* sp. from lesions), in addition to superinfection by different species of *Leishmania* [19,20,21,22,23]. The coexistence of *L. infantum* and *Crithidia* spp. in a patient with relapsed visceral leishmaniasis (VL) and nodular cutaneous manifestation, as reported in a case study by Rogerio et al. [24], implies that the co-infection scenario is associated with an atypical clinical presentation. Fakhar et al. [25] reported the occurrence of *Leishmania* spp. and *Crithidia* spp. in dogs in northeastern Iran in the context of co-infection in reservoir animals. Dario et al. [26] also described the occurrence of *Crithidia* infection in numerous mammalian species in Brazil. Another study found Culicoides mosquitoes to be potential vectors of *Leishmania* and *Crithidia* transmission in southern Thailand [27]. All of these studies provide context for various aspects of *Crithidia* within the spectrum of leishmaniasis, raising concerns about the impact of *Crithidia* infection in epidemiology, on clinical manifestations and resistance to treatments.

The present study describes a dye-based real-time quantitative PCR assay that utilizes two new specific targets to discriminate the species responsible for VL in Brazil (*L. infantum*) from other non-*Leishmania* species such as *Crithidia* sp., which can also be found in patients with VL [19,20,24,28]. The primer set for *L. infantum* was specifically designed for the LinJ31_2420 gene, which encodes the putative p-nitrophenylphosphatase enzyme belonging to the phosphatase family. This gene might be involved in biological processes such as phosphate metabolism, signal transduction, and nucleic acid synthesis [29,30]. The other target is the catalase gene, found only in monoxenous trypanosomatids [31,32]. In this context, we standardized and validated both targets for the detection and estimation of parasite load in tissues of VL patients, cultured parasites isolated from VL patients, domestic animal hosts (dogs and cats), samples from experimental infections (humans and hamsters), phlebotomine samples, and samples of an atypical and fatal case reported by Maruyama et al. [28]. These new targets can assist in the diagnosis of VL at research centers and in epidemiological surveillance within endemic areas of the disease. Furthermore, we provide novel molecular detection assays for parasite identification using qPCR, which can discriminate co-infections with other monoxenous trypanosomatid parasites, such as *Crithidia*-related species, reports of which have increased recently in humans [19,20,24,33,34,35,36].

## 2. Material and Methods

### 2.1. Search Strategy for New Gene Targets and Primer Design

Unique genes for *Leishmania* species were first retrieved from the genome analyses performed by Rogers et al. [37]. Target genes were reanalyzed using the search strategy tool available on TriTrypDB [38], in which search operations (intersect and/or minus) used BLASTN and Orthology (OrthoMCL algorithm) to combine results and retrieve information. The primer sets designed for *L. infantum* were based on the LinJ31_2420 gene (NCBI Reference Sequence: XM_001467522.1) (https://www.ncbi.nlm.nih.gov/gene/?term=XM_001467522.1; accessed on 26 July 2023). Primer sets for *Crithidia* sp. LVH60A were designed using genome assembly (GenBank assembly GCA_003671345.1). Oligonucleotides for targeted sequences were designed using the Primer-Blast tool [39], specifically checking parameters using the genomes of hosts (human, taxid:9609; dog, taxid:9615; cat, taxid:9685; rodents, taxid:9989). Primer specifications are listed in Table 1.

The sequences of catalase genes for *C. fasciculata* (reference CfCl) and the LinJ31_2420 gene by *L. infantum* (reference JPCM5) and *Leishmania* spp. were obtained from the TriTrypDB database [38]. Catalase genes for *Crithidia* sp. LVH60A were retrieved using local BLASTN searches (BLAST+ executables downloaded from NCBI). Additionally, BLASTN searches using amplicon and sequence primers were performed locally against downloaded genome sequences of *Crithidia* sp. LVH60A (https://www.ncbi.nlm.nih.gov/datasets/genome/GCA_030078075.1/, accessed on 25 May 2023), *C. fasciculata* reference Cf-Cl (https://www.ncbi.nlm.nih.gov/datasets/genome/GCA_000331325.2/; accessed on 22 October 2022), *L. infantum* reference JPCM5, Genome assembly ASM287v2 (https://www.ncbi.nlm.nih.gov/datasets/genome/GCF_000002875.2/; accessed on 22 October 2022), and *L. infantum* HUUFS14 (https://www.ncbi.nlm.nih.gov/datasets/genome/GCA_003671315.1/; accessed on 22 October 2022) with default parameters. Sequence searchers using amplicon and sequence primers against other Leishmaniinae species were performed using BLASTN, available in TriTrypDB resources.

### 2.2. DNA Extraction

Tissue DNA extraction was performed using the PureLink Genomic DNA Mini Kit (Invitrogen Life Technologies, Carlsbad, CA, USA), according to the manufacturer’s recommendations for the patients’ bone marrow and peripheral blood, as well as for the spleen and liver samples of dogs and hamsters. The genomic DNA from bone marrow aspirates and peripheral blood of cats with VL was obtained in a study by Vioti et al. [40] performed in the municipality of Ilha Solteira, São Paulo, Brazil, between 2015 and 2019.

Genomic DNA of the parasite cultures was extracted using the Wizard^®^ Genomic DNA Purification Kit (Promega, Madison, WI, USA) according to the manufacturer’s recommendations after 6–8 days (approximately 10^7^ parasites) for all parasite species. *Leishmania* strains (*L. infantum*: WHO/MHOM/BR/74/PP75 and HUUFS14; *L. amazonensis*: MHOM/BR/1973/M2269 and IFLA/BR/67/PH8; *L. braziliensis*: MHOM/BR/75/M2903, LTCP15171, and LTCP393) were maintained in vitro in Schneider’s Drosophila medium (GibcoTM, Paisley, SC, UK) supplemented with 20% fetal bovine serum (FBS) (GibcoTM, Grand Island, NY, USA), 1% penicillin–streptomycin–glutamine (GibcoTM, Carlsbad, CA, USA), 2% filtered male urine, and a final volume adjusted with incomplete Schneider’s Drosophila medium and incubated at 27 °C for 4–9 days for growth. *Crithidia* sp. isolates (LVH60_C3—clone 3; LVH117_C1—clone 1; and LVH120_C5—clone 5) were maintained in Schneider’s Drosophila medium supplemented with 10% FBS in an incubator at 25–27 °C for 4–7 days for growth. Trypomastigote *T. cruzi* (Y strain) culture was maintained in Novy–MacNeal–Nicolle medium [41] with liver infusion tryptose medium (LIT) [42] in an incubator at 27 °C for 7 days. Choanomastigote culture of *Crithidia fasciculata* (TCC039E strain) was maintained in Schneider’s Drosophila medium supplemented with 10% FBS and 1% penicillin–streptomycin–glutamine (GibcoTM, Carlsbad, CA, USA) in an incubator at 27 °C for 4–7 days. No difference in DNA extraction efficacy was observed, regardless of the parasite species or strain used.

DNA was extracted after in vitro infection using a Wizard^®^ Genomic DNA Purification Kit (Promega, Madison, WI, USA). In vitro infection assays were performed on THP-1 monocytes (a human cell line derived from acute monocytic leukemia), as described in Section 2.5.1. THP-1 cells were maintained in culture flasks (5 mL of complete RPMI 1640 with 10% FBS and 1% penicillin–streptomycin–glutamine, GibcoTM, Carlsbad, CA, USA), and the medium was changed every two days and maintained in an incubator at 37 °C and 5% CO_2_. For cell differentiation, monocytes were treated with 0.1 µM PMA (Phorbol 12-Myristate 13-Acetate) (Sigma-Aldrich, St. Louis, MO, USA) and incubated for 48 h at 37 °C and 5% CO_2_ to induce differentiation into adherent macrophages.

DNA of sandfly vectors was extracted using the Biopur Kit Extraction Mini Spin Plus (Biometrix, Pinhais, PR, Brazil), according to the manufacturer’s recommendations. All DNA samples were verified for purity (ratios 260/230 nm and 260/280 nm) using spectrophotometry (NanoVue, GE Healthcare, Björkgatan, Uppsala, Sweden) and quantified using Qubit Fluorometric Quantification (Invitrogen) with a Qubit™ dsDNA HS Assay Kit (Invitrogen). The DNA was stored at −20° C until use.

### 2.3. Real-Time Quantitative PCR (qPCR) Assays

qPCRs were performed using a fluorescent dye that binds to the double-stranded DNA molecule (qPCRBIO SyGreen Mix Separate-ROX, PCR BioSystems, Archway Rd, LDN, UK) and 0.2 µM of each primer (forward and reverse) in a final volume of 10 µL. The qPCRs also contained 12 ng of genomic DNA from human, parasite, macrophage cell line, phlebotomine, or animal samples.

The LinJ31_L42486.1 primer (Forward 5′-GCGACGTCCGTGGAAAGAA-3′/Reverse 5′-GGCGGGTACACATTAGCAGAA-3′) used elsewhere [43,44,45,46] was used as an external control for *L. infantum* in the in vitro assays.

Primers for endogenous genes were used for host DNA quality checks. The endogenous retrovirus group 3 (ERV-3) gene (forward 5′-CATGGGAAGCAAGGGAACTAATG-3′/reverse (5′-CCCAGCGAGCAATACAGAATTT-3′) [47,48] and glyceraldehyde 3-phosphate dehydrogenase (G3PDH) gene (forward 5′-TCAACGGATTTGGCCGTATTGG-3′/reverse 5′-TGAAGGGGTCATTGATGGCG-3′) [49,50] were used to amplify human and animal samples, respectively. Primers for the cacophonic gene (IVS6_ (forward 5′-GTGGCCGAACATAATGTTAG-3′/reverse 5′-CCACGAACAAGTTCAACATC-3′) [51] were used in the sandfly vectors.

The cycling conditions were as follows: 95 °C for 20 s for denaturation, followed by 40 cycles at 95 °C for 5 s, 60 °C for 30 s, and a melting curve at 95 °C for 15 s and 60–95 °C for 1 min (0.5 °C/5 s).

#### 2.3.1. Standard Curve

Parasite load was estimated using known amounts of genomic DNA extracted from promastigote cultures of *L. infantum* (HUUFS14 strain) and considering the DNA mass equivalent to one parasite. This calculation is detailed in the Supplementary Material Table S1.

The genome size of *L. infantum*, which is 32.13 Mb [52], was taken into account with 15.8% mass of kinetoplast (kDNA) of one parasite [53]. Standard curves were constructed using HUUFS14 strain DNA (*L. infantum*) with the LinJ31_2420 primer. Additionally, a standard curve was constructed using the Catalase_LVH60_12060_1F primer to identify *Crithidia* sp. [28] in DNA samples using the LVH60_C3 strain as a template.

The predicted genome length of the LVH60 clinical isolate was 34.4 Mb (available in the NCBI database under accession BioProject PRJNA940846). This size was used to estimate the DNA mass equivalent to that of a parasite [54]. Calculations of DNA mass equivalent to one parasite are shown in Supplementary Table S1. Serial dilutions corresponding to 10^5^ up to 1 parasite/reaction were used for the standard curve construction (in Supplementary Table S2). Host genetic material (human or animal) not infected by any trypanosomatid was used as a fixed background, containing 3 ng of DNA for each standard curve point.

#### 2.3.2. qPCR Assay Evaluation

DNA samples from *L. amazonensis* (M2269 strain), *Crithidia* sp. (LVH120 isolate, BioSample SAMN33579964), *L. braziliensis* (M2903 strain), *T. cruzi* (Y strain), and uninfected vertebrate hosts (human, canine, feline, and mouse) were used as negative controls to verify non-specific amplification.

Spike-in qPCR was performed by mixing different proportions of genomic DNA from each known species [55], to evaluate the accuracy of the LinJ31_2420 and Catalase_LVH60_12060_1F primers. The assay was performed in duplicate using a mixture of HUUFS14 strain DNA (*L. infantum*) and *Crithidia* sp. DNA (LVH60_C3 strain). Parasite proportions were calculated according to the mass equivalence of the parasite DNA (Supplementary Materials Table S3). A fixed background of 3 ng DNA from each host species was used.

Reproducibility and repeatability assays were performed in duplicate using two different qPCR devices: (i) a 7500 Fast Dx Real Time PCR Machine (Applied Biosystems, Thermo Fisher Scientific, Waltham, MA, USA), with data generated by 7500 Software Fast Real Time PCR Systems v2.0.6; and (ii) an AriaMx Real-Time PCR System (Agilent, Santa Clara, CA, USA), with data generated by AriaMx/AriaDx software. These assays were evaluated using the percentage coefficient of variation (CV) = [(standard deviation/mean) × 100], based on the mean Cq values [44,56].

### 2.4. PCR, Amplicon Sequencing, and Sequence Analysis

PCRs were performed using approximately 100 ng of promastigote DNA, 30 ng of human DNA, and 20 ng of animal DNA as a template, and a Taq DNA Polymerase kit (Thermo Scientific, Waltham, MA, USA). The reaction consisted of a final concentration of 1× Taq Buffer (750 mM Tris-HCl (pH 8.8), 200 mM (NH_4_)_2_SO_4_), 0.2 mM Deoxyrubonucleotides triophosphate (dNTP) (Thermo ScientificTM, Waltham, MA, USA), 0.4 µM of each primer (forward and reverse), 2 mM MgCl_2_, 0.05 unit of Taq DNA Polymerase, and nuclease-free water to a final volume of 25 µL per reaction. The cycle conditions for LinJ31seq and Crid2.1seq were maintained in a Biometra Tone (Analytikjena, Jena, Turíngia, DE) thermocycler at 95 °C for initial denaturation, followed by 34 cycles at 94 °C for 45 s, 55 °C for 30 s, 72 °C for 40 s, and 72 °C for 5 min for final extension. The cycle conditions for LVH60a_Tig001 were maintained in the same thermocycler at 95 °C for 20 s for denaturation and amplification at 95 °C for 5 s, followed by 40 cycles at 60 °C for 30 s, 72 °C for 1 min, and then 72 °C for 5 min for final extension.

All genomic material used in the PCRs with LinJ31seq, Crid2.1seq, and LVH60a_Tig001 primers was visualized by agarose gel electrophoresis (1%), and images were documented using Chemidoc XR (BioRad, Hercules, CA, USA). The patient and dog samples required enrichment of the product from the first PCR using the LinJ31seq primer. PCR enrichment consisted of using the diluted product (at a 1:10 ratio) of the first PCR using the LinJ31seq primer (see Table 1) in a second reaction with the same primer. The same procedure was performed for sandfly samples using the LinJ31_2420 primer.

Genomic DNA of bone marrow (patients and dogs) and clinical isolates was used in Nested-PCRs with the TRY927 primer (Forward 5′-GAAACAAGAAACACGGGAG-3′/Reverse 5′-CTACTGGGCAGCTTGGA-3′) and SSU561 primer (Forward 5-TGGGATAACAAAGGAGCA-3′/Reverse 5′-CTGAGACTGTAACCTCAAGC-3′) in the study by Noyes et al. [57] to amplify a small subunit ribosomal RNA (18S rRNA) region, using a Biometra Tone thermocycler (Analytik Jena, Jena, Turíngia, DE). Amplicons were purified using the PureLink™ PCR Purification Kit (Invitrogen) and quantified by spectrophotometry using NanoVue apparatus (GE Healthcare, Björkgatan, Uppsala, Sweden). Amplicon sequencing was performed using the Sanger method with the BigDye^®^ Terminator v3.1 Cycle Sequencing Kit (Applied Biosystems, Foster City, CA, USA) in an Applied Biosystems 3130 Genetic Analyzer. The reactions were prepared with 3.2 pmol of the forward primer SSU561, 10–20 ng of purified PCR product of SSU561 amplicons, and ultrapure water adjusted to a final volume of 7 µL.

Phylogenetic analysis of 18S rRNA sequences was performed using MEGA6 [58]. Phylogenetic trees with sequences from an LVH60 patient and from the bone marrow and peripheral blood of dogs were reconstructed using the Maximum Likelihood (ML) method based on the Tamura–Nei model with Bootstrap tests (1000 replicates). Corresponding 18S rRNA sequences from other trypanosomatid species to compose the analysis were retrieved from the NCBI database and/or TriTrypDB using BLASTN searches.

### 2.5. Experimental Validation

#### 2.5.1. In Vitro Infection with THP-1 Macrophages

The detection and estimation of the parasitic load in in vitro infections were performed using qPCR assays. For infections using the HUUFS14 strain (*L. infantum*), the LinJ31_2420 primer was used and the LinJ31_L42486.1 primer [43,44,45,46] was used as an external control. In addition, in vitro infections with *Crithidia* sp. LVH60A, LVH117C1, and C. fasciculata (TCC039E strain) were quantified through qPCR using the Catalase_LVH60-12060 primer.

In vitro infection was performed in 12-well plates for DNA extraction. Immortalized THP-1 monocytic cells (a human cell line derived from acute monocytic leukemia) [59] were differentiated into macrophages and infected with different species/strains of parasites. Monocytes were counted such that each well contained 1 × 10^6^ cells. A 24-well plate was also used for microscopy, in which 10 mm round coverslips were placed for microscopy analysis. The monocytes were seeded at a density of 5 × 10^5^ cells/well.

The stationary phases of *Leishmania infantum* (HUUFS14 strain), *Crithidia* sp. strains (LVH60a_C1 and LVH117_C1), and *C. fasciculata* (TCC039E strain) obtained on the 7–8th day of culture were used for in vitro infection. The ratio used for the infection of THP-1 cells was 10 parasites to 1 macrophage (MOI 10:1). Initially, the plates were incubated at 37 °C and 5% CO_2_ for 3 h. After this period, all wells were washed with incomplete RPMI 1640 (GibcoTM, Grand Island, NY, USA) to remove extracellular parasites, and complete medium was added to the infected cells. Infected cells were incubated for 24, 48, and 72 h. The Romanowsky staining method was used with the Rapid Panoptic Kit (LarboClin, Pinhais, PR, BR) on 10 mm round coverslips, which were then mounted on slides. The microscopic evaluation of infected macrophages was performed manually using an optical microscope (Olympus, Tokyo, TYO, JPN, 100× objective), counting 100 macrophages per coverslip and determining the number of amastigotes in these cells.

#### 2.5.2. In Vivo Infection

Male hamsters (*Mesocricetus auratus*) were separated into groups of four individuals at 3 weeks of age and infected by intracardiac inoculation with a concentration of 1.7 × 10^7^ metacyclic promastigotes/mL of *L. infantum* in the stationary growth phase, previously selected with peanut agglutinin (PNA) (500 µg/mL). Clinical isolates were obtained from patients with VL at the Institute of Tropical Diseases Natan Portela in Teresina, Piauí, Brazil, and were identified as strains #1580, #1686, #1851, and #515. PBS 1× was used as a negative control (non-infected group) for infection in hamsters. The animals were followed up for 9 months and euthanized with ketamine (200 mg/kg) and xylazine (10 mg/kg), based on clinical signs of the disease. During necropsy, the spleen and liver were excised and segmented for DNA extraction.

#### 2.5.3. Sample Collection

Tissue samples (bone marrow and peripheral blood) and clinical isolates (LVH abbreviation) were obtained from patients diagnosed with VL at the Federal University Hospital of the University of Sergipe (HUUFS) in Aracaju, Sergipe, Brazil. Clinical isolates used in the in vivo experimental infections (Section 2.5.2.) were isolated from patients with VL admitted to the Institute of Tropical Diseases, Natan Portela, Teresina, Piauí, Brazil. A positive rK39 serological test (Kalazar Detect Rapid Test, InBios International Inc., Seattle, WA, USA) along with direct observation of *Leishmania* in a bone marrow aspirate or a positive culture in Novy–MacNeal–Nicolle (NNN) medium were used to confirm the diagnosis of VL. Bone marrow and peripheral blood samples were collected from dogs and cats diagnosed with VL; diagnosis of the animals was based on the characterization of the signs of leishmaniasis and on the hematological, biochemical, and serological parameters shown by the animals housed in a shelter in the municipality of Ilha Solteira, São Paulo, Brazil. Phlebotomine samples (*Lutzomiya longipalpis*) were collected as described by Ferreira et al. [60], between January and June in 2014 and 2016 in the municipality of Rio Verde de Mato Grosso, Mato Grosso do Sul, Brazil. The pools of insects collected consisted of 10–15 females captured in CDC light traps placed in the peridomicile. The insects were stored in 70% alcohol until use.

### 2.6. Data Analysis

Amplicon sequencing: The chromatograms generated by Sanger sequencing were subjected to quality control using Chromas software Version 2.1.5 (https://technelysium.com.au/wp/, accessed on 30 April 2022). Data analysis was performed using the Mega6 alignment and phylogeny program [58].

Parasite detection: The detection and efficiency of the LinJ31_2420 primer in *L. infantum* (HUUFS14 and PP75 strains) were compared with another set of established primers, LinJ31_L42486.1 [43,44,45,46], by analyzing the amplification plots (Cq values) and dissociation curves. *Crithidia* sp. parasites were also detected using the Catalase LV60-12060_1F primer. Primer efficiency analyses were performed using amplification plots and the dissociation curve graphs. The detection limit of the qPCR assay, which refers to the smallest amount of parasite detected, was determined by calculating the mean and standard deviation of the Threshold Cycle (Cq) values [61,62].

Statistical analyses were performed using GraphPad Prism Version 5. Two-way ANOVA was performed using the mean values of the estimated parasite burden for in vitro infections with THP-1 macrophages. Statistical significance was set at *p* < 0.05.

### 2.7. Ethics Statement

The procedures for the collection of material from patients were performed according to the guidelines of the Brazilian Human Research Ethics Evaluation System (CEP/CONEP) and approved by the local Ethics Committee: (i) Federal University of Sergipe, Aracaju, Sergipe, Brazil (protocol CAAE:04587312.2.0000.0058) and (ii) Institute of Tropical Diseases Natan Portela in Teresina, Piauí, Brazil (protocol CAAE:0116.0.045.203-05). Prior to the study, patients or their legal guardians were informed and signed the consent form.

Animal samples (dogs and cats) were collected according to the guidelines of the Animal Use Ethics Committee (CEUA) of the Animal Science and Food Engineering Faculty of the University of São Paulo (FZEA-USP) and were approved under registration number 6147100518. The genomic DNA samples from cats with VL used in this study were the same as those used by Vioti et al. [40] between 2015 and 2019 from two animal shelters in the municipality of Ilha Solteira, São Paulo, Brazil. This study was approved by the CEUA of the Faculty of Food Science and Engineering, University of São Paulo (FZEA-USP), under protocol numbers 7627010517 and 8541011019.

The procedures for infections in hamsters (*Mesocricetus auratus*) were approved by the Local Ethics Committee for the use of animals at the Ribeirão Preto Medical School of the University of São Paulo, CEUA-FMRP/USP (process number 234/201). The phlebotomine vectors from the study by Ferreira et al. [60] were collected from sites designated by the entomological surveillance of the municipality of Rio Verde de Mato Grosso, authorized by the System of Authorization and Information on Biodiversity (SISBIO) under registered license number 33156-2.

## 3. Results

### 3.1. Real-Time PCR Performance and Validation of the Method

**Figure 1:**
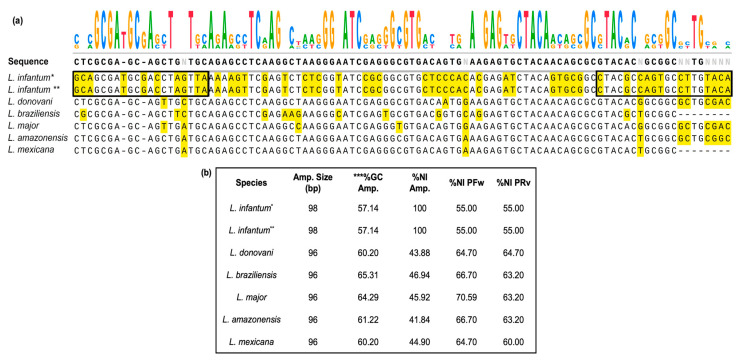
Sequence alignment of the region (98 bp) targeted by the primer LinJ31_2420 in *Leishmania* parasites. (**a**) Region of 98 bp in the putative p-nitrophenylphosphatase gene, in which the LinJ31_2420 primer aligned in the *L. infantum* JPCM5 reference strain (XM_001467522.1) and in the HUUFS14 laboratory strain. Positions at the 5′ and 3′ ends where primer annealing occurs are highlighted in the black box. The sequence logo of the alignment is shown at the top of the figure. Positions with nucleotide polymorphisms are highlighted in yellow. (**b**) Box summarizes the main features of the sequence targeted by the LinJ31_2420 primer in *L. infantum* and other *Leishmania* species. The asterisks indicate: * JPCM5, the reference genome of *L. infantum* (Genome assembly ASM287v2) and ** HUUFS14, the genome of laboratory strain (Genome assembly HUUFS14) used for experiments. *L. donovani* (accession number: LdBPK 312410.1), *L. braziliensis* (accession number: LbrM. 31.2620), *L. panamensis* (accession number: LPAL13 310028600), and *L. major* (accession number: LmjF. 31.2340); *L. amazonensis* (accession number: LAMA 000645300) and *L. mexicana* (accession number: LmxM. 30.2340). Amp: amplicon; NI: percentage of nucleotide identity; *** percent guanidine (%G) and cytosine (%C); forward primer (PFw); reverse primer (PRv); bp: base pair. The alignment was generated and formatted using SnapGene^®^ software version 7.0.

First, we standardized a new gene target for the molecular detection of *L. infantum*. LinJ31_2420 (98 bp) and LinJ31_seq (444 bp) amplicons refer to the putative single-copy p-nitrophenylphosphatase gene, which is located on chromosome 31 of the JPCM5 reference genome [37,53,63]. When the first version of the genome was released, this gene was considered unique to *L. infantum*. Subsequently, it was also found in other *Leishmania* species (Supplementary Table S4). The alignment of primer sequences in the genes from the JPCM5 and HUUFS14 *L. infantum* strains showed 100% nucleotide identity (NI) for the putative p-nitrophenylphosphatase gene (NCBI Reference Sequence: XM_001467522.1). Primer sequence alignment in other *Leishmania* species showed lower and diversified nucleotide identity (NI) compared with *L. infantum*, in addition to two gaps in the region of the forward primer. In addition, the predicted melting temperature (Tm) for possible amplicons in other *Leishmania* species, as well as the GC content, differed from that observed for *L. infantum* (Figure 1). This means that if amplification of another *Leishmania* species putatively occurs, analysis of the dissociation curves may elucidate species identity.

For technical validation, qPCR assays were performed and compared with known primers described elsewhere [43,44,45,46], which were used for *L. infantum* detection (LinJ31_L42486.1). According to the analysis performance of the dissociation curve for LinJ31_2420 and LinJ31_L42486.1, single peaks were observed with Tm values of 83.35 °C and 77.56 °C, respectively (Supplementary Figure S1a,b), indicating the specificity and stability of primers. Amplification plots of six-point standard curves for both primers are shown in Supplementary Figure S1c,d. Detailed calculations for the standard curve construction are presented in Supplementary Table S2.

We then standardized the molecular detection of *Crithidia* sp. LVH60A. The catalase _LVH60-12060_1F primer was based on the predicted catalase enzyme gene identified in the genome of a clinical isolate of a *Crithidia*-related parasite (LVH60a clone 1 strain) obtained from an atypical VL case [28]. Catalase is absent in dixenous trypanosomatids (*Leishmania* and *Trypanosoma*) and is present in the monoxenous genera of Trypanosomatidae, such as *Crithidia* and *Leptomonas* [31,32,64] (Supplementary Table S5). This primer showed specificity for the *Crithidia* sp. LVH60A cultured isolate without amplification of *L. infantum* (HUUFS14 strain), *T. cruzi* (Y strain), or host DNA (human) (Supplementary Figure S2).

Six-point standard curves using the Catalase_LVH60-12060_1F primer with DNA samples from the *Crithidia* sp. LVH60-C1 strain and *C. fasciculata* TCC039E were generated (Supplementary Figure S3). Both *Crithidia* parasites showed similarities in terms of amplicon melting temperature, with a mean Tm of 84.79 °C (LVH60A) and 85.00 °C (TCC039E). The dissociation curve for TCC039E (*C. fasciculata*) exhibited a secondary peak at ~81 °C. To rule out the possibility of non-specific amplicons, we performed agarose gel electrophoresis analysis, which showed single qPCR products for all reactions (Supplementary Figure S3d). This dissociation curve profile can be explained by multi-stage melting transitions raised by sequence-specific characteristics, such as polymorphisms and GC rich regions.

**Figure 2:**
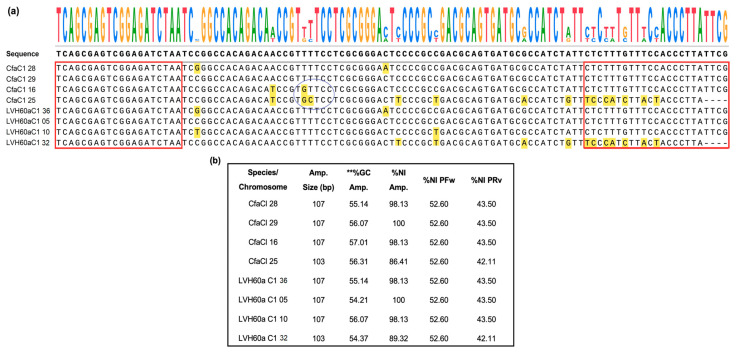
Sequence alignment of the region (107 bp) targeted by the primer Catalase_LVH60-12060_1F in *Crithidia* parasites. (**a**) Region of 107 bp in the putative catalase gene, where Catalase_LVH60-12060_1F primer alignment sequences occur in LVH60a_C1 (*Crithidia* sp., LVH60A) and *C. fasciculata.* The positions at the 5′ and 3′ ends, where primer annealing occurs, are highlighted in the red frame. The sequence logo of the alignment is shown at the top of the figure. The blue circle indicates the regions of polymorphisms found in CfaCl 16 and CfaCl 25 from *C. fasciculata*. (**b**) The box summarizes the main features of the sequence targeted by the Catalase_LVH60-12060_1F primer in the *Crithidia* parasites. Four copies of the gene in these species were observed in the chromosome of *C. fasciculata* (CfaCl 28 CFAC1_280006600, CfaCl 29 CFAC1_290005500, CfaCl 16 CFAC1_160031400, and CfaCl 25 CFAC1_250006200) and sequences regions of genome of the LVH60A_C1 (Chromosome 36, Chromosome 05, Chromosome 10, and Chromosome 32. Amplicon (amp.); percent nucleotide identity (%NI); ** percent guanidine (%G) and cytosine (%C); forward primer (PFw); reverse primer (PRv); base pair (bp). Alignment was generated and formatted using SnapGene^®^ software version 7.0 (Insightful Science).

The sequence of the amplicon Catalase_LVH60-12060_1F (107 bp) was searched against the genome of *Crithidia* sp. LVH60A and *C. fasciculata* (CfCl strain). BLASTN match hits revealed four copies of the catalase gene in both species located on different chromosomes (Figure 2). In both genomes, three copies were of the same size and one copy was of a smaller size (103 bp). The four copies presented few changes in sequence compared with the reference amplicon (depicted in yellow); however, *C. fasciculata* catalase copies presented three more polymorphisms than *Crithidia* sp. LVH60A. These variations in sequences due to several gene copies may explain the potential occurrence of secondary peaks in qPCR melting curves.

qPCR standard curves for parasite detection and quantification were set up with known quantities of DNA from promastigote culture in 10-fold serial dilutions and a fixed host DNA quantity as background. Parasite quantity for the standard curve was estimated by calculating the equivalent genomic DNA mass for one parasite (~81 fg) set up at six points ranging from 1 × 10^5^ parasites to 10^0^ parasites (Table S2, Supplementary Material). Amplification plots and standard curves for reactions using LinJ31_2420 or Catalase_LVH60-12060_1F primers showed reliable performance (Figure S4 and Figure S5, respectively; Supplementary Material).

To evaluate the specificity, repeatability, and reproducibility of the qPCRs using both primers, host DNA was used as the background (simulating an infected host sample), and assays were performed on qPCR instruments from two different brands to assess the technical variation. Regarding primer specificity for detecting parasite material, genomic DNA from four different types of mammalian hosts was used as the background in qPCR standard curve reactions: human, dog, cat, and mouse. All of these types of hosts are commonly used in leishmaniasis research. Regardless of the source of host DNA as background, all qPCR standard curve reactions resulted in high performance in assays running on qPCR instruments of two different brands, as observed in parameters such as the coefficient of determination (R^2^) and efficiency (Eff) displayed in Table S6 (Supplementary Material), showing that there was no cross-amplification of host DNA. Coefficient R^2^ values for LinJ31_2420 and Catalase_LVH60-12060_1F primers ranged from 0.95 to 0.99, with very low variation among different types of host DNA background. Reactions performed on one particular instrument showed the highest values of R^2^ for both primers (~0.99).

**Figure 3:**
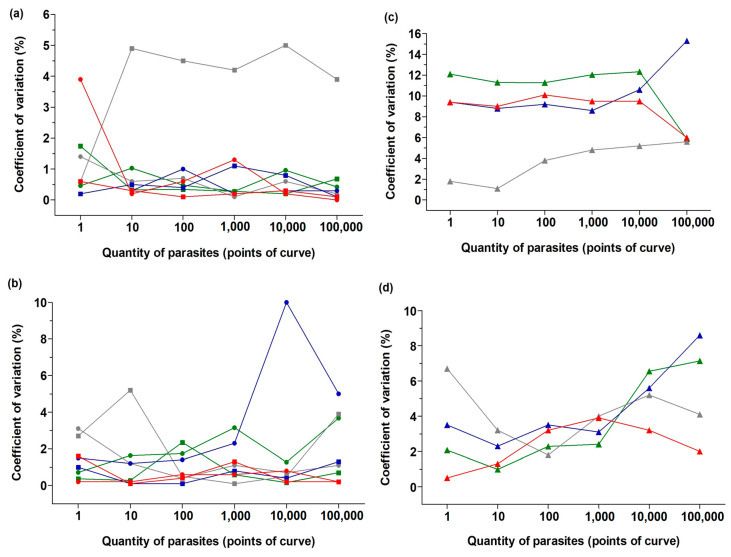
Coefficient of variation (%) of qPCR standard curves in intra- and across inter-assays: (**a**) Repeatability (intra-variation in graphs on the left) and reproducibility (inter-variation in graphs on the right) of assays with the LinJ31_2420 primer (**a**,**c**) and assays with Catalase_LVH60-12060_1F primer (**b**,**d**), measured using the coefficient of variation (CV%) expressed as a percentage. CV% of intra-assays was calculated with replicates within the same plate (run with instrument 1, square symbols, or run with instrument 2, circle symbols), whereas the CV% of inter assays was calculated with replicates across plates performed with different qPCR instruments (i.e., comparison between plates/instruments, triangle symbols). DNA from the M2903 strain (*L. braziliensis*), M2269 strain (*L. amazonensis*), Y strain (*T. cruzi*), and vertebrate hosts did not show amplification for the primers LinJ31_2420 and Catalase_LVH60-12060_1F. Amplification was not observed in the no-template reaction. Control endogenous genes of endogenous retrovirus group 3 (ERV-3) for humans and glyceraldehyde 3-phosphate dehydrogenase (G3PDH) for other vertebrate hosts were used. DNA from vertebrate hosts was used as a fixed background (6 ng) at all points in the qPCR curve (green indicates human DNA, gray indicates cat DNA, blue indicates dog DNA, and red indicates mouse DNA). Graphs were generated using GraphPad Prism Version 5.00 software.

The repeatability and reproducibility of qPCRs were assessed through CV measurements in intra-assays (reactions in the same plate) and inter-assays (reactions from different plates were run on different instruments). Overall, the assays presented reliable performance, i.e., they exhibited very low coefficients of variation (Figure 3). Intra-assays using the LinJ31_2420 primer presented a homogenous average CV as low as 1.5%, as observed in Figure 3a (except for reactions using cat DNA as the background, CV ~5%). For the catalase primer, the variation in intra-assays was slightly higher but still exhibited small values, as shown in Figure 3b. Reactions using human DNA and dog DNA showed the highest variation (CV ~2.5% and CV ~3.5%, respectively).

Comparisons between assays set up using different qPCR instruments also presented a low inter-assay CV with both primers across different amounts of parasite DNA (Figure 3c,d). For the Lin31_2420 primer, the detection of *L. infantum* DNA presented an average CV of ~10% between assays performed with two instruments, using human, dog, and mouse DNA as the host background; cat DNA presented the lowest CV. Variations in the catalase primer used to detect *Crithidia* sp. were lower than 10% regardless of the host background; however, a small fluctuation in the CV values across the points of the standard curve was observed, mainly in the extreme number of parasites in the reactions (1 and 10^5^ parasites).

**Figure 4:**
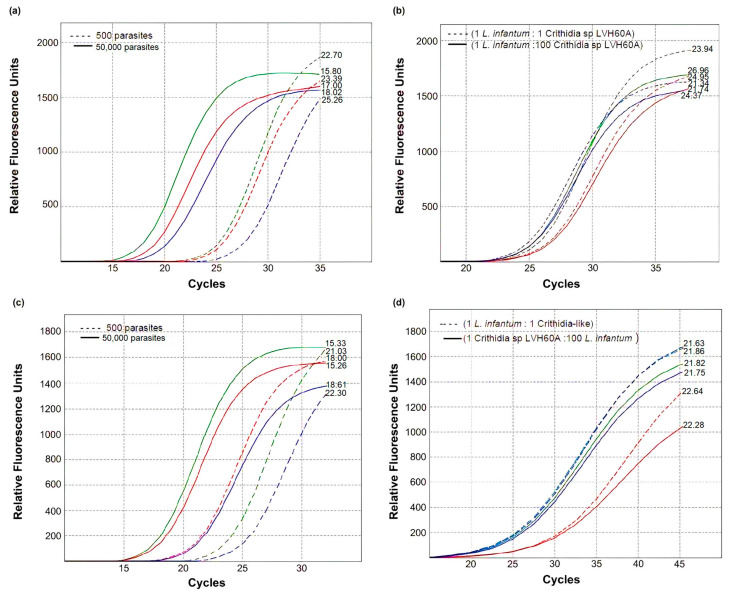
Accuracy of qPCR assays on parasite quantification assessed through a spike-in experiment for each primer. (**a**) Accuracy of qPCR assays for parasite quantification assessed through a spike-in experiment for each primer. (**a**) Quantification of *L. infantum* DNA with the LinJ31_2420 primer using known amounts of equivalent number of parasites (500 parasites, curves in dashed lines; 50,000 parasites, curves in solid lines). The numbers on the curves indicate the threshold Cq values for quantification of *L. infantum*. (**b**) Quantification of *L. infantum* DNA with the LinJ31_2420 primer in a spike-in experiment using DNA from *Crithidia* sp. LVH60A (LVH60_C3 strain) in two proportions: 500 *L. infantum* parasites:500 *Crithidia* sp. LVH60A parasites (1:1), curves in dashed lines; and 500 *L. infantum* parasites:50,000 *Crithidia* sp. LVH60A parasites (1:100), curves in solid lines. The numbers in the curves indicate the threshold Cq values for quantification of *L. infantum*. (**c**) Quantification of *Crithidia* sp. LVH60A DNA with the Catalase_LVH60-12060_1F primer using known amounts of an equivalent number of parasites (500 parasites, curves in dashed lines; 50,000 parasites, curves in solid lines). The numbers on the curves indicate the threshold Cq values for quantification of *Crithidia* sp. LVH60A. (**d**) Quantification of *Crithidia* sp. LVH60A DNA with the Catalase_LVH60-12060_1F primer in a spike-in experiment, using DNA from *L. infantum* in two proportions: 500 *Crithidia* sp. LVH60A parasites:500 *L. infantum* parasites (1:1), curves in dashed lines; and, 500 *Crithidia* sp. LVH60A parasites:50,000 *L. infantum* parasites (1:100), curves in solid lines. The numbers on the curves indicate the threshold Cq values for the quantification of *Crithidia* sp. LVH60A. DNA (6 ng) from each vertebrate host was used as a background to simulate an infection (graphs (**a**,**c**) or co-infection scenario (graphs (**b**,**d**), in which green, blue, and red curves represent host DNA from humans, dogs, and mice, respectively.

Recently, coinfection with *L. infantum* and *Crithidia* sp. was reported in a multirelapsed case of visceral leishmaniasis [24]. To simulate a co-infection scenario, we performed spike-in assays, in which known amounts of *Crithidia* sp. LVH60A and *L. infantum* DNA were mixed experimentally, and different host DNA was used as the fixed background in the reactions (Figure 4). For reference, assays with pure parasite DNA (either from *L. infantum* or *Crithidia* sp.) were set up with 5 × 10^2^ or 5 × 10^4^ equivalent numbers of parasites. The observed Cq values for the Linj31_2420 primer, using *L. infantum* DNA as a template, were within the expected value for a 10-fold serial dilution (qPCR slope ~3.29, Table S6), with a difference lower than half Cq. As observed in Figure 4a, the Δ Cq between reactions containing 5 × 10^4^ (Cq ~17) and 5 × 10^2^ (Cq ~24) equivalent numbers of parasites were ~6.8 cycles, as expected. Similarly, this small difference was observed in assays using the catalase primer with *Crithidia* sp. LVH60A DNA as a template, using only human as the host background, as displayed in Figure 4b. The Δ Cq values between reactions containing 5 × 10^4^ and 5 × 10^2^ equivalent numbers of parasites for dog and mice as the host background were ~3.2 cycles rather than ~6.4 cycles, as would be expected according to the qPCR slope for the catalase primer. Furthermore, we spiked *L. infantum* DNA into the reactions to detect *Crithidia* sp. LVH60A, and vice versa (we spiked *Crithidia* sp., LVH60A DNA into reactions to detect *L. infantum*) with different proportions of spiked parasite DNA amounts, 1:1 (5 × 10^2^ *L. infantum* to 5 × 10^2^ *Crithidia* sp. LVH60A), and 1:100 (5 × 10^2^ *L. infantum* to 5 × 10^4^ *Crithidia* sp. LVH60A or vice versa). In this artificial co-infection simulation, both primers were very specific, presenting very low inhibition in the presence of DNA from another parasite species, regardless of the proportion tested, as shown in Figure 4c,d for LinJ31_2420 (Cq ~23.4) and catalase (Cq ~21.9) primers, respectively.

### 3.2. Validation of the Novel qPCR Assay and Gene Targets in Experimental Conditions

**Figure 5:**
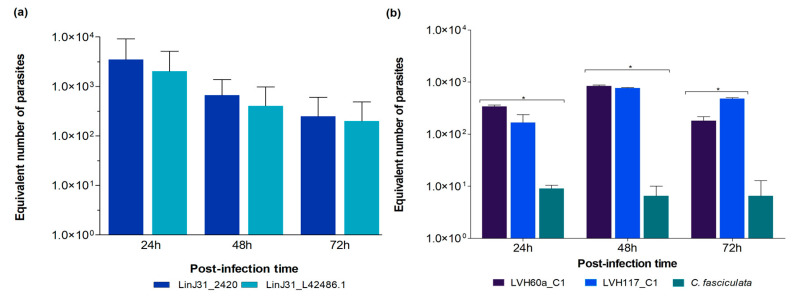
Quantification of *L. infantum* or *Crithidia* parasites in in vitro infections of THP-1-derived macrophages. (**a**) DNA extracted from THP-1 cultures infected with HUUFS14 *L. infantum* strain at 24, 48, and 72 h was used to quantify the equivalent number of parasites through qPCR using LinJ31_2420 primer. Primer LinJ31_L42486.1 [43,44,45,46] was used for comparison. No statistically significant difference was found between the two groups. (**b**) DNA extracted from THP-1 cultures infected with LVH60a_C1 and LVH117_C1 *Crithidia* sp. LVH60A strains at 24, 48 and 72 h, as well as the TCC039E *C. fasciculata* strain, were used to quantify the equivalent number of parasites by qPCR using the Catalase_LVH60_12060_1F primer. Two-way ANOVA was performed using GraphPad Prism 5.00; *p* < 0.05 was considered statistically significant; * *p* < 0.001.

To assess the performance of these novel qPCR assays in quantifying parasites experimentally, we performed the in vitro infection of human macrophages derived from THP-1 cells with *L. infantum* or *Crithidia* sp. LVH60A parasites (LVH60a_C1 and LVH117_C1, both are clones) [24,28]. The parasite load was estimated at different post-infection time periods, such as 24, 48, and 72 h for *L. infantum* infection (Supplementary Figure S7a); parasite quantification was also performed with the LinJ31_L42486.1 primer as a reference control (Figure 5a). The standard curve with the LinJ31_2420 primer presented average slope values of −3.26 ± 0.06 with R^2^ of 0.94 ± 0.03, and an efficiency of 103.8%, while the standard curve with the LinJ31_L42486.1 primer presented an average slope of −3.03 ± 0.06, R^2^: 0.97 ± 0.03 and 117.09% of efficiency. Control reactions for human DNA detection were performed using the ERV-3 primer, which is only able to detect the macrophage DNA (12 ng of DNA from the in vitro infection experiment, presented an average Cq of 26 ± 0.05). As shown in Figure 5a, both primers for *L. infantum* were able to quantify parasite load at different time points, showing a high positive correlation between the two measures (Supplemental Figure S6, R^2^ = 0.99). The parasite loads for *L. infantum* at 24 h, 48 h, and 72 h were ~2766, ~493, and ~241 equivalent parasites, respectively.

Although *Crithidia* species are supposedly monoxenous parasites exclusive to insects and are considered unable to infect mammalian cells, we showed, for the first time, that *Crithidia* sp. LVH60A parasites (cultured isolates LVH60a_C1 and LVH117_C1) [24,28] can infect human macrophages (Supplementary Figure S7b,f). The standard curve with the catalase primer presented an average slope of −3.41 with an R^2^ of 0.97 ± 0.002 and an efficiency of 96.2%. The parasite load of *Crithidia* sp. LVH60A was also calculated at different post-infection times, and assays showed an average of 463 equivalent parasites in 12 ng of macrophage DNA (Figure 5b), which roughly corresponded to 18,000 human cells. As a reference control for negative infectiveness, we used the *C. fasciculata* TCC039E strain for the set of in vitro infections (Supplementary Figure S7c). As shown in Figure 5b, very low numbers of *C. fasciculata* parasites were detected, regardless of the time of infection (~10 parasites per 18,000 cells). We observed a significant difference between the LVH60a_C1 and TCC039E strains and a non-significant difference (ns) between LVH60a_C1 and LVH117_C1 at 24 h post-infection. A significant difference was observed between the LVH60a_C1 and TCC039E strain and between LVH117_C1 and TCC039E at 48 h post-infection, as well as a significant difference between the LVH60a_C1 and LVH117_C1 strains, and the LVH117_C1 and TCC039E strains at 72 h post-infection. The parasite load of *L. infantum* was much higher than that of *Crithidia* sp., LVH60A; however, it is noteworthy that both LVH60a and LVH117 strains were able to infect human cells in vitro (Supplementary Figure S7f).

**Figure 6:**
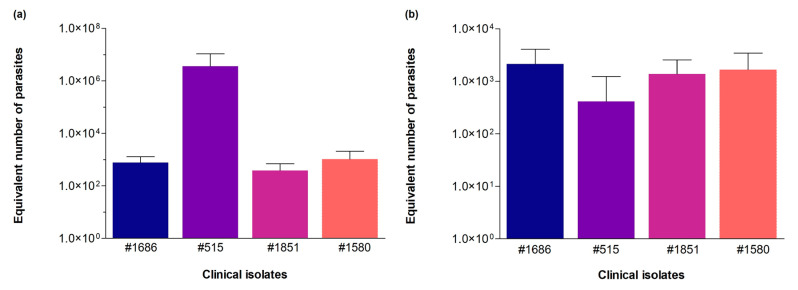
Quantification of *L. infantum* in organs of hamsters (*Mesocricetus auratus*) subjected to experimental in vivo infections with clinical isolates of *Leishmania*. The clinical isolates were coded as #1851, #1686, #1580, and #515. (**a**) Parasitic load in the liver and (**b**) parasitic load in the spleen, assessed by qPCR using the LinJ31_2420 primer. Equivalent number of parasites/reaction (i.e., in 20 ng of host DNA). The parasitic load among the isolates observed in each organ did not show statistically significant differences in the number of parasites. Kruskal–Wallis test was performed using GraphPad prism 5.00 software.

We also tested the novel qPCR assay to estimate parasite load in samples of in vivo infection with *L. infantum* clinical isolates from an endemic region (Teresina, Piauí, Brazil). Experimental infections were performed in hamsters using the cultured isolates #515, #1580, #1686, and #1851. DNA was extracted from liver and spleen tissues. A standard curve with the LinJ31_2420 primer was constructed using the HUUFS14 strain (*L. infantum*) with an average slope of −3.106, linearity coefficient R^2^ of 0.966, and an efficiency of 109.88%. Control reactions for hamster DNA detection were performed using the G3PDH primer (20 ng of DNA from the spleen and liver presented average Cq values of 20.32 ± 0.12 and 20.22 ± 0.05, respectively). In vivo infection with *L. infantum* clinical isolates showed ~727 equivalent parasites infecting the liver (Figure 6a) and ~1725 equivalent parasites infecting the spleen (Figure 6b) for the cultured isolates #1686, #1851, and #1580 (in 20 ng of DNA). We highlight cultured isolate #515, which presented more 1000-fold more parasites in the liver of hamsters, showing the high proliferative capacity of this strain in this tissue.

### 3.3. Validation of the Novel qPCR Assay and Gene Targets through Parasite Detection in Clinical Isolate Cultures and Host Tissue Samples

Before qPCR primers for the novel gene targets were tested in clinical and animal samples, we standardized primers for conventional PCR to screen samples for qPCR analysis. Primers were designed to be exclusive to *L. infantum* (LinJ31seq), *Crithidia* sp. LVH60A, and/or *C. fasciculata* (Crid2.1seq). In the absence of the necessary resources to develop a qPCR experiment, these primers can be used to identify these species using conventional PCR.

Crid2.1seq amplicon (502 bp) refers to a hypothetical protein gene found specifically in *Crithidia* sp. LVH60A and *C. fasciculata*. Very small variations in GC content, amplicon size, and NI were observed between *Crithidia* sp. LVH60A and *C. fasciculata* (Table 2). The Crid2.1seq primer was suitable for PCR using purified parasite DNA from *Crithidia* sp. LVH60A clinical isolate cultures. No amplification was observed for *L. infantum* DNA (HUUFS14 and PP75 strains), neither *L. braziliensis* (M2903, LTCP15171, and LTCP393 strains), *T. cruzi* (Y strain) or human DNA from THP-1 cell line (Supplementary Figure S8a). 

The LVH60a_Tig001 amplicon (128 bp) refers to a hypothetical protein gene found specifically in *Crithidia* sp. LVH60A. This primer was suitable for PCR using parasite DNA purified from cultures of *Crithidia* sp. and LVH60A clinical isolates that showed no amplification of *C. fasciculata* (TCC039E strain), as shown in Figure S8b (Supplementary Material).

The primers LinJ31seq and Crid2.1seq, designed for *L. infantum* and *Crithidia* sp., respectively, were used to screen samples of VL patients and clinical isolates by PCR (Table 3). In addition, the SSU rRNA (18S) region was amplified by Nested PCR [57] followed by amplicon sequencing using the Sanger method (Supplementary Tables S7 and S8). The accession numbers of the small subunit rRNA (ssrRNA) sequences used in this analysis are listed in Table S9.

Due to the very low amount of parasite DNA in patient samples (bone marrow and peripheral blood), LinJ31seq amplicon visualization on agarose gel electrophoresis was possible after performing a second batch of PCR with LinJ31seq primer using the 10-fold diluted PCR product for enrichment of the target sequence (Supplementary Figure S9). Thirteen of the seventeen bone marrow samples were PCR-positive for LinJ31seq (*L. infantum*). Then, parasite detection and estimation of the parasite load in positive samples were performed through qPCR using LinJ31_2420 (*L. infantum*) and Catalase_LVH60-12060_1F (*Crithidia* sp. LVH60A).

**Figure 7:**
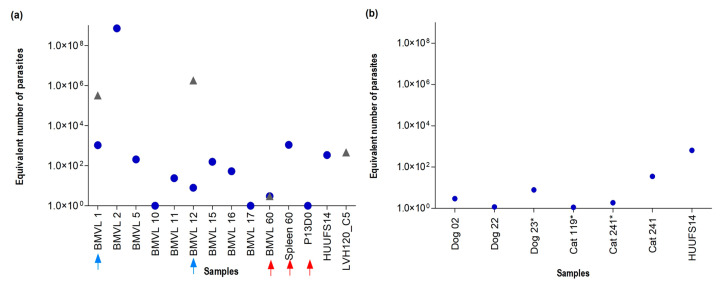
Quantification of *L. infantum* and *Crithidia* parasites in samples of VL patients and domestic animals by qPCR. (**a**) Equivalent number of parasites quantified in clinical samples of bone marrow aspirates (BMVL), spleen, and peripheral blood (P13D0) from patients with VL. Detection of *L. infantum* and/or *Crithidia* was performed using LinJ31_2420 (blue circles) and Catalase_LVH60_12060_1F (gray triangles) primers. Blue arrows indicate co-infection of *L. infantum* with *Crithidia* sp. LVH60A parasites. Red arrows refer to patient samples from a fatal case reported by Maruyama et al. (2019) [28]. (**b**) Equivalent numbers of parasites were quantified in bone marrow aspirates and peripheral blood (*) from dogs and cats with clinical signs of VL and a positive serological test for *Leishmania*. Only *L. infantum* was detected in domestic animal samples. DNA from *L. infantum* (HUUFS14 strain) and *Crithidia* sp. LVH60A (LVH120_C5 strain) was used as the positive control for qPCR.

In determining the parasitic load with the LinJ31_2420 primer, the construction of the standard curve consisted of six points corresponding to 1 × 10^1^ to 1 × 10^6^ equivalent number of parasites in 40.7 ng of parasite DNA (average Cq 18.05 ± 1.17) at 1 × 10^1^ equivalent number of parasites in 0.0407 pg of parasite DNA (average Cq 32.36 ± 0.27) showed a slope of −3.071, R^2^ of 0.949, and 111.6% of efficiency. The detection limit in bone marrow samples that matched the 10 equivalent parasites in 6 ng DNA was identified to be in a Cq range of 30.0 ± 0.8. For some samples, it was possible to detect one (1) parasite (1 × 10^0^) in 6 ng DNA with an average Cq of 34.3 ± 0.8. Interestingly, bone marrow aspirates of BMVL1 and BMVL12 tested positive for the two primers (Figure 7a), suggesting that the patients were co-infected with *L. infantum* (CqBMVL1 25.25; CqBMVL12 31.96) and *Crithidia* sp. LVH60A (CqBMVL1 16.55; CqBMVL12 12.04). Notably, BMVL1 and BMVL12 presented a much higher parasite quantification of *Crithidia* sp. LVH60A than *L. infantum*.

Additionally, we tested samples from atypical and fatal VL cases reported by Maruyama et al. [28] using Catalase_LVH60-12060_1F and LinJ31_2420 primers. *L. infantum* was detected in spleen and blood (Figure 7a). A bone marrow aspirate sample (BMVL60) tested positive for *Crithidia* sp., LVH60A, and *L. infantum*. To support the results of the LVH60 case, the SSU rRNA (18S) marker was sequenced from these samples. Amplicon sequence analysis revealed that samples from the liver and spleen matched with *L. infantum*, whereas samples from skin biopsy, bone marrow, and blood matched with *Crithidia* sp. LVH60A (Supplementary Figure S10). Overall, these results confirmed the hypothesis raised by others [63] that the patient was co-infected with *L. infantum* and *Crithidia*.

Both the LinJ31seq and Crid2.1seq primers were tested in samples from dogs diagnosed with canine visceral leishmaniasis (VL, bone marrow, and peripheral blood), as well as in samples from cats displaying symptoms related to feline VL. No amplification was observed using the Crid2.1seq primer, and only LinJ31seq PCR products were visualized in dog samples. The results for *L. infantum* were supported by Sanger sequencing of the SSU rRNA (18S) marker [58] (Supplementary Material, Figure S11). The accession numbers of small subunit rRNA (ssrRNA) sequences used in phylogenetic analysis are shown in Table S9. The qPCR assays for parasite detection corroborated the PCR results, showing that only *L. infantum* was detected in the tested samples (Figure 7b).

The LinJ31_2420 amplicon for vector samples proved to be viable for detection and was used in qualitative qPCR assays. There was no amplification for conventional PCR using the LinJ31seq primer and no amplifications for the Catalase-LVH60_12060_1F primer. DNA samples from seven sandfly pools were used; all females of the species *Lutzomyia longipalpis* were positive for *L. infantum*, as confirmed by Sanger sequencing of the internal transcribed spacer region, according to the results of the study by Ferreira et al. [60]. Detection was possible through the enrichment by PCR with LinJ31seq. Detection was made possible through pre-enrichment using conventional PCR with LinJ31seq. The DNA used in this first study was ~12 ng, and the PCR product was used for the qPCR with LinJ31_2420 for the detection of protozoan material, showing Cq values for the vector sample #10 of 20.54 ± 0.49, vector #63 of 19.65 ± 0.33, vector #67 of 17.87 ± 0.007, vector #75 of 28.51 ± 0.44, vector #119 of 33.09 ± 0.36, vector 126 of 27.49 ± 0.007, and vector I7 of 24.91 ± 0.05, as shown by the amplification graph in Figure S12 in the Supplementary Material. The quality and integrity of the insect DNA were verified with the cacophonic gene region (IVS6) [51] present in sandflies, and in *Lutzomiya longipalpis*, the average Cq and standard deviation were 24.8 ± 5.1 and Tm 82.40 °C.

## 4. Discussion

Diagnostic strategies for VL developed in recent decades include parasitological and serological tools and procedures to detect protozoans in vertebrate host samples [10,16]. However, these technologies lack sensitivity and specificity, which is crucial for disease therapy and prognosis. With the advent of molecular biology tools, there has been a focus on the more robust and accurate detection, identification, and quantification of protozoan genetic material in host samples [64].

There are some reports and clinical case studies of patients with leishmaniasis in which clinical isolates have been identified and are closely related to species of trypanosomatids of the genus *Leptomas* [33,34,35] and *Crithidia* [20,24,28,36,65]. A review of the study and research of monoxenic trypanosomatids in clinical findings of vertebrate hosts carried out by Boucinha et al. [19] revealed that the cases seen so far were questionable by only focusing on known pathogenic trypanosomatids, since the routine diagnoses (parasitological and serological) used for the determination of the disease cannot describe the causative species. The phylogenomic applications and evolutionary studies of these kinetoplastids are being developed to understand the distribution of the biology, diversity, and establishment of the life cycle of these protozoa belonging to the polyphyletic group classified as Crithidiatae [66]. Kraeva et al. [67] addressed the need to evaluate additional samples from VL patients using specific targets capable of detecting monoxenic trypanosomatids in clinical samples. As discussed in the review by Boucinha et al. [19], an appropriate tool to ascertain whether a monoxenic trypanosomatid infection occur in leishmaniasis should ideally use PCR techniques.

In this study, we designed and standardized PCR and qPCR assays using species-specific target sequences for *L. infantum* (LinJ31_2420 and LinJ31seq), as well as for the detection of *Crithidia* (Catalase_LVH60-120_1F and Crid2.1seq) in experimental and clinical samples, as an alternative for the molecular screening and investigation of VL cases, such as those studied by Maruyama et al. [28]. Pereira et al. [68] showed the importance of using qPCR in the screening of blood donors living in areas endemic to VL in Brazil, identifying the presence of protozoan DNA in these donors. We demonstrated that through genome-based calculations of these parasites, it is possible to estimate the parasitic load and detect traces of infections in clinical samples (Figure 7).

In standard curves performed in serial dilutions based on the calculation of the mass DNA equivalent to a parasite, we used a fixed amount of DNA from non-infected hosts as the background in the reactions, which showed no interference in the performance of the assays. In a study by Jara et al. (2013) [56], the detection and quantification of parasitic load by qPCR in samples of skin lesions and mucocutaneous lesions were also evaluated using higher amounts of human DNA. Sundarshan et al. [11] confirmed that quantification given by the standard curve is a relative measurement compared with clinical samples, i.e., the number of protozoa measured in a sample/tissue does not exactly match the actual parasitic load of the individual, as factors such as the stage of infection in which the host is found and losses in the DNA extraction process can generate a non-robust quantification. The primer LinJ31_2420 proved to be species-specific for VL samples and can be useful for estimating parasite load, both quantitatively and qualitatively in a range, such as high, intermediate, or low loads. The primer did not present amplification using material from other *Leishmania* species, *Crithidia* sp. LVH60A, *C. fasciculata*, and hosts (dogs, cats, rodents, and phlebotomines).

The Catalase LVH60-12060_1F primer, which targets the catalase gene, was specific for *Crithidia* sp. LVH60A and *C. fasciculata* and did not produce cross amplification for *Leishmania* sp. and host DNA. Analysis of the amplicon in genomic sequences of *Crithidia* sp., LVH60A, and *C. fasciculata* CfCl reference strain (Figure 2) revealed four gene copies with slight differences in nucleotide composition. According to Weirather et al. [69], dye-based tests can discriminate species through a dissociation curve, which is influenced by the GC content of the amplicon. However, these tests need to be carefully evaluated to avoid misidentification of species, as variations in melting temperature due to differences in buffer components, concentration of template DNA, and experiments on different devices can cause confusion [69,70,71].

When simulating a co-infection scenario between *L. infantum* and *Crithidia* sp. in spike-in assays with known amounts of pure DNA from each parasite species and then mixed together with host DNA, we observed that the Cq values were within the expected range (Figure 4), indicating the accuracy of the assay. The decrease in amplification efficiency observed in the results of the spike-in assay with DNA from dogs and mice may be related to the host DNA. This interference may be more pronounced if the amount of host DNA in the reaction is relatively high, which may be due to human failure in the dilutions. Overall, both primers when performing spike-in DNA showed reliable specificity with low inhibition in the presence of DNA from other parasite species, regardless of the proportion tested. Therefore, the results of the spike-in assay indicated that both primers were able to detect DNA from *Crithidia* sp. LVH60A and *L. infantum* in a co-infection scenario.

In this study, we present specific targets that discriminate parasite protozoa present in clinical samples of VL. We suggest the use of PCR with primer sets LinJ31seq and Crid2.1seq, as well as qPCR with LinJ31_2420 and Catalase-LVH60-12060_1F for parasite identification. It is important to consider the targets chosen for qPCR diagnosis, opting for species-specific genes such as those described here. For instance, the use of kDNA as a target may offer non-robust data due to the heterogeneity present in the minicircles, and the number of copies varies between kinetoplastids. In addition, the use of constitutive genes as targets, such as SSU rDNA [72], cannot precisely discriminate the species. For instance, a primer based on the 18S rRNA region may cross-amplify with other trypanosomatids, as described by Filgueira et al. [73].

Studies have reinforced the importance of molecular methods in becoming a reliable alternative in the diagnostic routine for leishmaniasis, especially for cases in which the patient has a low parasite load. This will enable rapid identification of the protozoan species causing infections and determination of treatment options. [69,74]. Our study focused on parasite detection using a dye-based method, which is cheaper than probe-based method but implies the need for several singleplex reactions to identify each suspect species, which is a drawback. Multiplex qPCR systems enable the simultaneous detection of multiple genes in a single reaction being advantageous because they are less time-consuming; however, they require extensive protocol optimization and expensive probes. The targets studied here can be future be exploited by others for the development of a multiplex system.

Parasite load in bone marrow samples from patients with VL was successfully estimated using LinJ31_2420 and Catalase-LVH60_12060_1F primers with a limit of detection estimated in 1 × 10^1^ parasites in 6 ng of DNA (Cq 30.0 ± 0.8). Using the Catalase-LVH60_12060_1F primer, it was possible to detect and estimate the number of parasites in samples BMVL1, BMVL12, and BMVL60, indicating the presence of two species in these samples, *L. infantum* and *Crithidia* sp. LVH60A (Figure 7).

Recently, Rogerio et al. [24] reported a case of severe VL in a male child patient, in which co-infection of *L. infantum* and *Crithidia* sp. LVH60A was observed. Here, we used the same clinical isolate culture, the LVH117 strain (isolated from bone marrow), to demonstrate the ability of this novel parasite to infect human macrophages through the in vitro infection of THP-1 cells (Figure 5b and Supplementary Figure S7f). The BMVL60 sample refers to the case reported for the first time in a study by Maruyama et al. [28], in which it was coded as LVH60. Through phylogenetic analysis using small subunit rRNA (ssrRNA) sequences (Figure S10, Supplementary Material), we observed that this patient was co-infected with *L. infantum* and *Crithidia* sp. LVH60A. It was possible to corroborate this co-infection using qPCR with LinJ31_2420 and Catalase-LVH60_12060_1F primers (Figure 7a). Co-infection with monoxenic trypanosomatids in patients with leishmaniasis has been reported in studies [20,33,34,35,67,75,76] since the 1980s. To clarify the frequency of these cases and their association with treatment resistance and epidemiology in leishmaniasis, new cases should be researched in depth, and further examination of the likely function of these monoxenous trypanosomatids in the pathology of disease should be performed.

In conclusion, using the molecular approach presented in this study, we standardized and evaluated new species-specific targets for *L. infantum* and *Crithidia* sp. LVH60A parasites with the ability to detect and estimate parasite load in samples from different hosts involved in infection (human, animal, and experimental) and can be used for epidemiological studies, parasite load monitoring, and therapeutic follow-up. For diagnostics, these primers can be helpful, predominantly for the elucidation of VL cases of co-infection with *Crithidia* parasites. Finally, all investigations that place *Crithidia* within the context of the leishmaniasis spectrum need to be further addressed, raising questions about the influence of *Crithidia* infection on epidemiology, clinical symptoms, and treatment resistance.

## Figures and Tables

**Table 1:** General information about primers.

Primer Name	Primer F (5′-3′)	Primer R (5′-3′)	Amplicon Size	Usage
LinJ31seq	GTGCACGCCAATGTCTTTGT	GCCCATGGTTGAGCTAGGTT	444 bp	Detection of *L. infantum* by PCR
LinJ31_2420	GCAGCGATGCGACCTAGTTA	TGTACAAGGCACTGGCGTAG	98 bp	Detection and quantification of *L. infantum* by qPCR
Crid2.1seq	TCACTTTGGCGGTATCAGTG	GCATCAGCTGACCCTTTCTC	502 bp	Detection of *Crithidia* sp. LVH60A and *C. fasciculata* by PCR
LVH60a_Tig001	GTTAGAGCGACTAGCCCGTG	GGGTAGAGGAGAGAGGTGGG	128 bp	Detection of *Crithidia* sp. LVH60A by PCR
Catalase_LVH60-12060_1F	TCAGCGAGTCGGAGTCTAA	CGAATAAGGGTGGAAACAAAGAG	107 bp	Detection and quantification of *Crithidia* sp. LVH60A and *C. fasciculata* by qPCR

F, forward; R, reverse; bp, base pair.

**Table 2:** Characterization of Crid2.1seq primers in *Crithidia* sp. LVH60A, *C. fasciculata*, and *Leptomonas* spp. sequences.

Primer	Species	Chr.	Gene ID or Access Number *	Amp. ** GC%	Amp. Size	Percent Nucleotide Identity
Crid2.1seq	*Crithidia fasciculata* (CfCl)	16	CFAC1_160022800	67.47%	501 bp	A = 87.0 T = 76.0 C = 162.0 G = 176.0
	*Crithidia* sp. *LVH60A* (LVH60a)	10	CP119667.1	67.13%	502 bp	A = 86.0 T = 79.0 C = 166.0 G = 171.0
	*Leptomonas pyrrhocoris* (H10)	15	LpyrH10_15 ^a^	No match		
		16	LpyrH10_16 ^b^			
	*Leptomonas seymouri* (ATCC_30220)	48	Lsey_0048 ^a^	No match		
		01	Lsey_01 ^b^			

* TriTryDB; ** guanidine (G) and cytosine (C); ^a^ Forward sequence. ^b^ Reverse sequence. Adenine (A), timine (T) guanidine (G) and cytosine (C). Chr: chromosome; Amp: amplicon.

**Table 3:** Samples from VL patients and clinical isolates screened through PCR with novel primers for the SSU rRNA (18S) marker.

	Tissue	Number of Samples	PCR + LinJ31seq	PCR + Crid2.1seq	Nested-PCR TRY927/SSU561 [57]
Patient samples	BMVL ^a^	18	13	0 ^c^	18
	PB	14	6	0	7
Clinical isolates	CI ^b^	53	14	48	46
	Skin (nodule/papule)	4	2	2	4 ^c^
	Spleen	1	0	1	1 ^d^

^a^ BMVL: bone marrow aspirate visceral leishmaniasis; ^b^ CI: clinical isolates, i.e., parasite cultures obtained from bone marrow aspirate of visceral leishmaniasis patients; ^c^ PCR products with size smaller than expected were considered negative. PB: peripheral blood; GenBank accession number OQ581236.1 and OQ581229.1; ^d^ GenBank accession number OQ581233.1; ^c^/^d^ from Rogerio et al. [24].

## Data Availability

All data supporting the study findings are included in this published article or in the Supplementary Material. Amplicon sequences (partial 18S rRNA) were deposited in GenBank (https://www.ncbi.nlm.nih.gov/genbank/, accessed on 26 July 2023) under accession numbers OR228484-OR228495 and OR250364-OR250401, and in the European Nucleotide Archive (https://www.ebi.ac.uk/ena/, accessed on 26 July 2023) under accession numbers PRJEB25557 and PRJEB25906.

## Supplementary Materials

The following supporting information can be downloaded at: https://www.mdpi.com/article/10.3390/tropicalmed8080405/s1. Figure S1: The dissociation curve and amplification plot were generated using the LinJ31_2420 and LinJ31_L42486.1 primers. Figure S2: Specificity of the Catalase_LVH60-12060_1F primer. Figure S3: qPCR assay with the Catalase_LVH60-12060_1F primer pair. Figure S4: Standard curve with DNA from the HUUFS14 strain (*L. infantum*) with the LinJ31_2420 primer. Figure S5: Standard curve with DNA from the LVH60_C3 strain (*Crithidia* sp. LVH60A) with the Catalase_LVH60-12060_1F primer. Figure S6: Pearson’s correlation of post-infection times with HUUFS14 (*L. infantum*) using primers LinJ31_2420 and LinJ31_L42486.1. Figure S7: THP-1 macrophages infected with the HUUFS14 strain (*L. infantum*), LVH60a_C1, LVH117_C1 (*Crithidia* sp. LVH60A), and TCC039E (*C. fasciculata*). Figure S8: Agarose gel electrophoresis (1%) of amplifications with Crid2.1seq and the LVH60a_Tig001 primer. Figure S9: Electrophoresis in agarose gel (1%) of conventional PCR performed with samples of LV patients from HUUFS. Figure S10: Phylogenetic analyses of small subunit rRNA (ssrRNA) sequences from LVH60 patient samples. Figure S11: Phylogenetic tree with TRY927/SSU561 rRNA sequences detected in bone marrow and peripheral blood samples of dogs with VL. Figure S12: Amplification plot with the LinJ31_2420 primer in sandfly samples determined by qPCR. Table S1: Calculations of DNA mass equivalent to one parasite. Table S2: Serial dilution for the qPCR standard curve according to the calculation of the DNA mass equivalent to one parasite of *L. infantum* and *Crithidia* sp. LVH60A; Table S3: Table of proportions of the number of parasites for the spike-in assay; Table S4: Information of the gene ID, chromosome, location genome, and the number of copies of p-nitrophenylphosphatase putative gene in *Leishmania* spp. Table S5: Information of the gene ID, chromosome, location genome, number copies of the catalase gene in *Crithidia* sp., LVH60A (LVH60a), *C. fasciculata*, and *Leptomonas* spp. Table S6: Coefficient values for qPCR standard curves using host DNA as the background for the reactions. Assays were performed in two qPCR instruments of different brands with curves set up at six points in 10-fold serial dilutions. Table S7: PCR results with species-specific primers and molecular typing by Sanger sequencing. Table S8: PCR results with LinJ31seq and Crid2.1seq and molecular typing by Sanger sequencing of clinical isolates. Table S9: Accession numbers of small subunit rRNA (ssrRNA) sequences used in phylogenetic analysis are displayed in Supplementary Figures S8 and S9.
