# Supplementary material for: Parasite Detection in Visceral Leishmaniasis Samples by Dye-Based qPCR Using New Gene Targets of Leishmania infantum and Crithidia
Source: Trop Med Infect Dis. 2023 Aug 8;8(8):405. doi: 10.3390/tropicalmed8080405 (PMC10457869; doi:10.3390/tropicalmed8080405)
Supplement: Supplementary file 1 [file tropicalmed-08-00405-s001.zip › tropicalmed-2509720 - Supplemental-Figures_Takamyia-et-al-NTT2.pdf]

Supplementary Figures

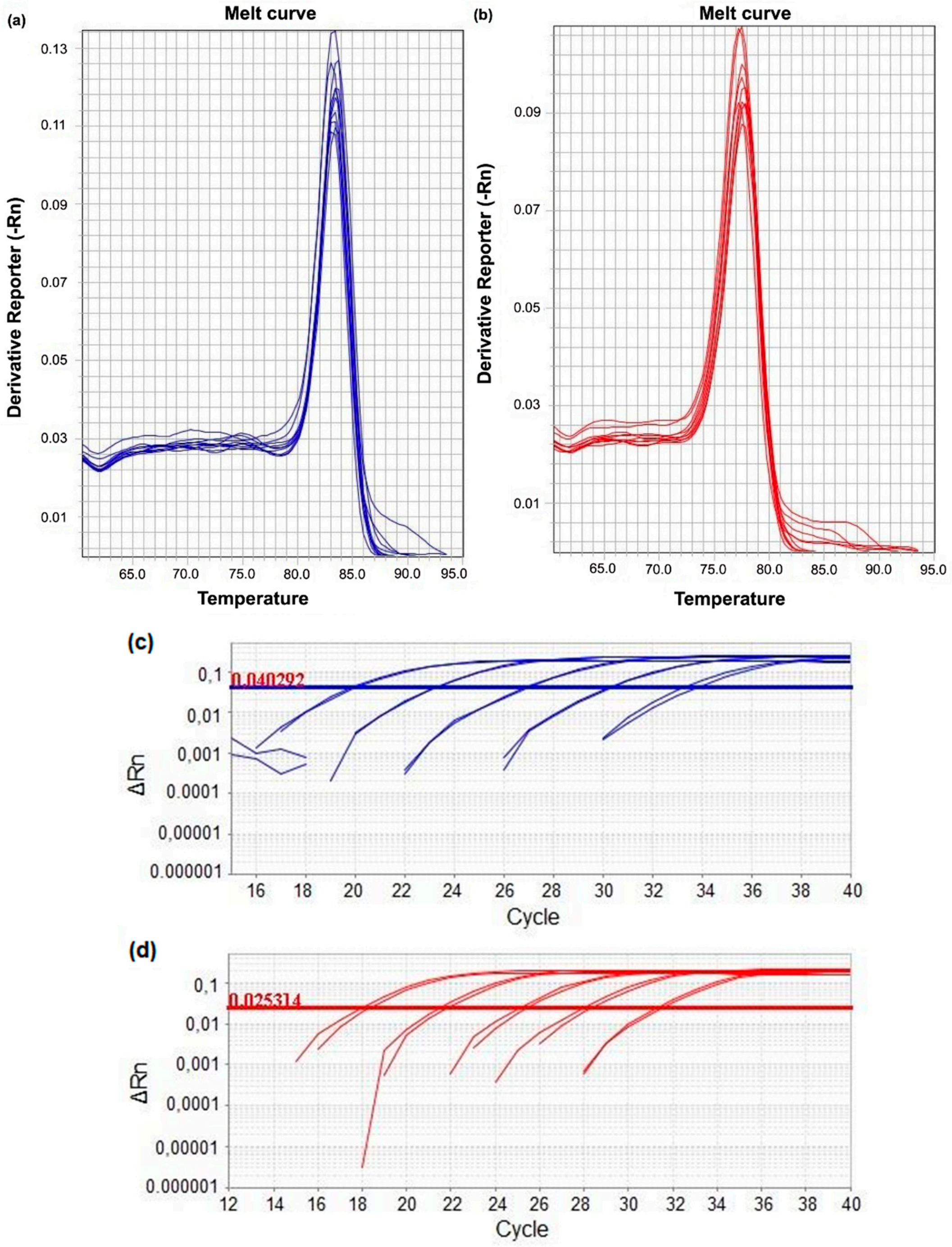

Figure S1: The dissociation curve and amplification plot were generated using the LinJ31_2420 and LinJ31_L42486.1 primers. (**a**) Dissociation curve for LinJ31_2420 primers exhibited an average Tm of 83.35°C. (**b**) Primers LinJ31_L42486.1 presented a dissociation curve with an average Tm of 77.56°C. DNA template from *L. infantum* HUUFS14 strain. **(c)** Amplifications with LinJ31_2420 utilized serial dilutions of L. infantum DNA (HUUFS14 strain) ranging from 40.7e-09 g/µL to 40.7e-15 g/µL. **(d)** The same approach was applied for the LinJ31_L42486.1 primer

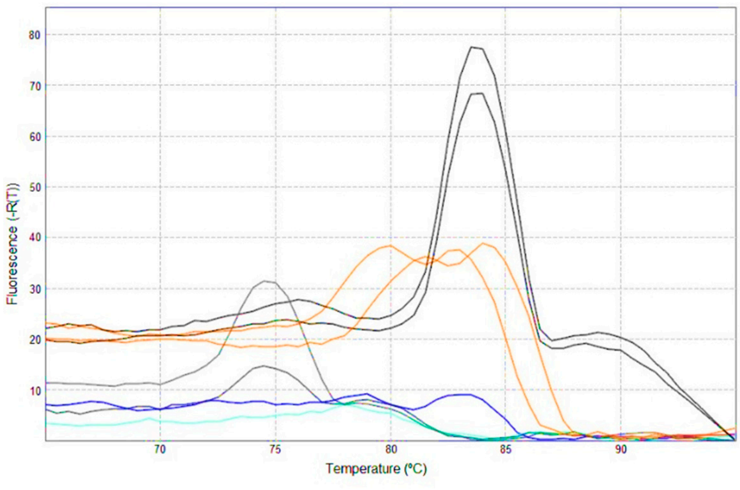

Figure S2: Specificity of the Catalase_LVH60-12060_1F primer. Dissociation curve shown with LVH60_C3 (*Crithidia-like*) (black curve) reaching Tm of 84.50°C. Not indicating specificity with human DNA material (orange curve), HUUFS14 strain (*L. infantum*) (gray curve) and *T. cruzi* (Y strain) (blue curve).

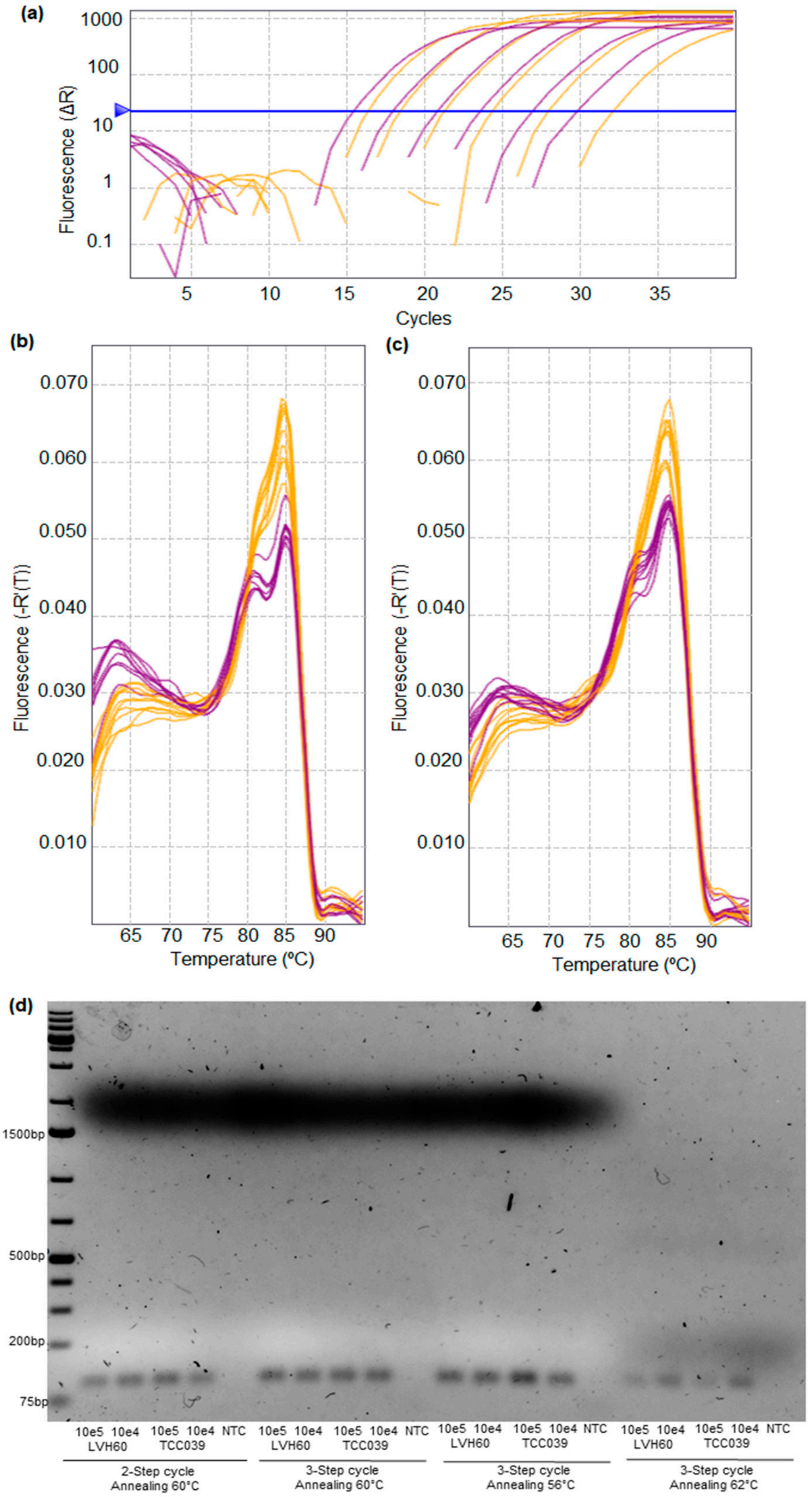

Figure S3: qPCR assay with Catalase_LVH60-12060_1F prime pair. (**a**) Amplification plot of six-points standard curve with Catalase_LVH60-12060_1F primer used 10-fold serial dilutions of genomic DNA from LVH60_C3 strain (*Crithidia sp* LVH60A) ranging from 43.6 e-9 g to 43.6 e- 15 g (in yellow) and DNA from TCC039E strain (*C. fasciculata*) ranging from 52.4 e-9 g to 52.4 e-

15 g (in purple); (**b**) Dissociation curves with DNA template from LVH60_C3 *Crithidia sp* LVH60A strain (yellow curves) showed an average Tm of 84.79°C, whereas reactions using DNA template from TCC039E *C. fasciculata* strain (purple curves) showed an average Tm of 85.00°C by 2- step cycle annealing at 60°C. (**c**) Dissociation curve C by 3- step cycle annealing at 60°C, DNA template from LVH60_C3 *Crithidia sp* LVH60A strain (yellow curves) showed an average Tm of 84.71°C, whereas reactions using DNA template from TCC039E *C. fasciculata* strain (purple curves) showed an average Tm of 85.00°C. (**d**) Agarose gel electrophoresis (1%) was performed to analyze the amplifications of the Catalase primer pair LVH60-12060_1F.

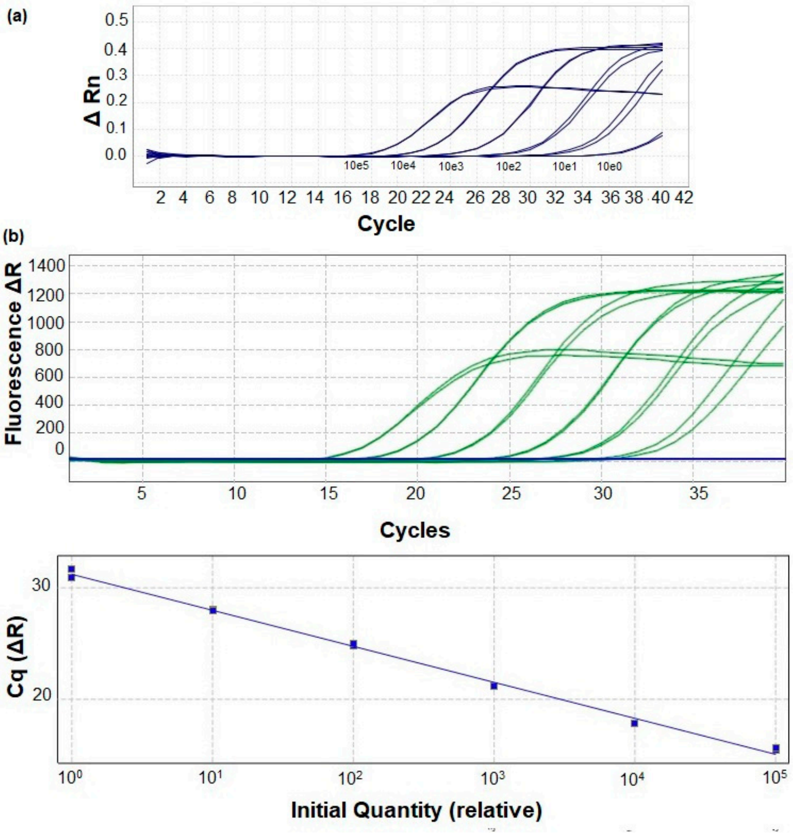

Figure S4: Standard curve with DNA from HUUFS14 strain (*L. infantum*) with LinJ31_2420 primer. (a) Amplification plot of the standard curve generated by apparatus one. (b) Amplification plot the standard curve generated by apparatus two. (c) standard curve each blue dot on the line represents the equivalent of from $1x10^5$ parasites to 10^0 parasites by apparatus two. Curve constructed according to the calculation of the equivalent mass of a parasite together with the DNA of the host not infected by trypanosomatids [6ng] as a fixed background of the reaction.

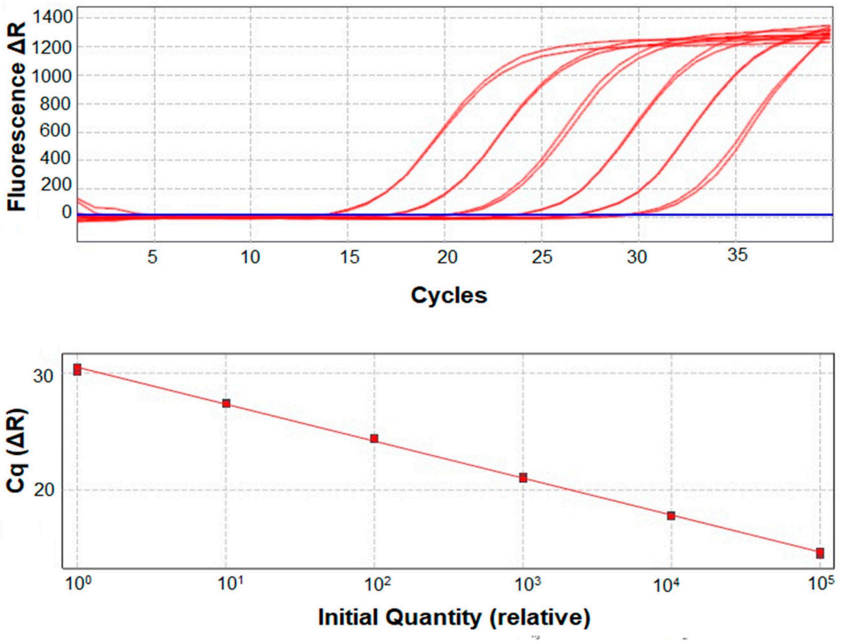

Figure S5: Standard curve with DNA from LVH60_C3 strain (*Crithidia* sp LVH60A) with Catalase_LVH60-12060_1F primer. Amplification plot of the standard curve and standard curve each red dot on the line represents the equivalent of from $1x10^5$ parasites to 10^0 parasites generated by apparatus two. Curve constructed according to the calculation of the equivalent mass of a parasite together with the DNA of the host not infected by trypanosomatids [6ng] as a fixed background of the reaction.

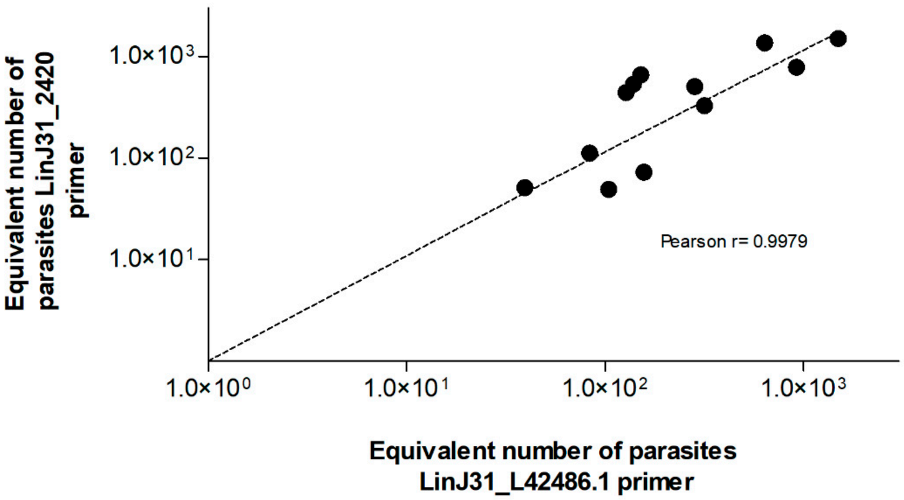

Figure S6: Pearson's correlation of post-infections time with HUUFS14 (*L. infantum*) was used primers LinJ31_2420 and LinJ31_L42486.1. High positive correlation between the two measures LinJ31_2420 and LinJ31_L42486.1 primers Pearson's correlation coefficient (r2= 0.9979). Software-generated data GraphPad Prism Version 5.00.

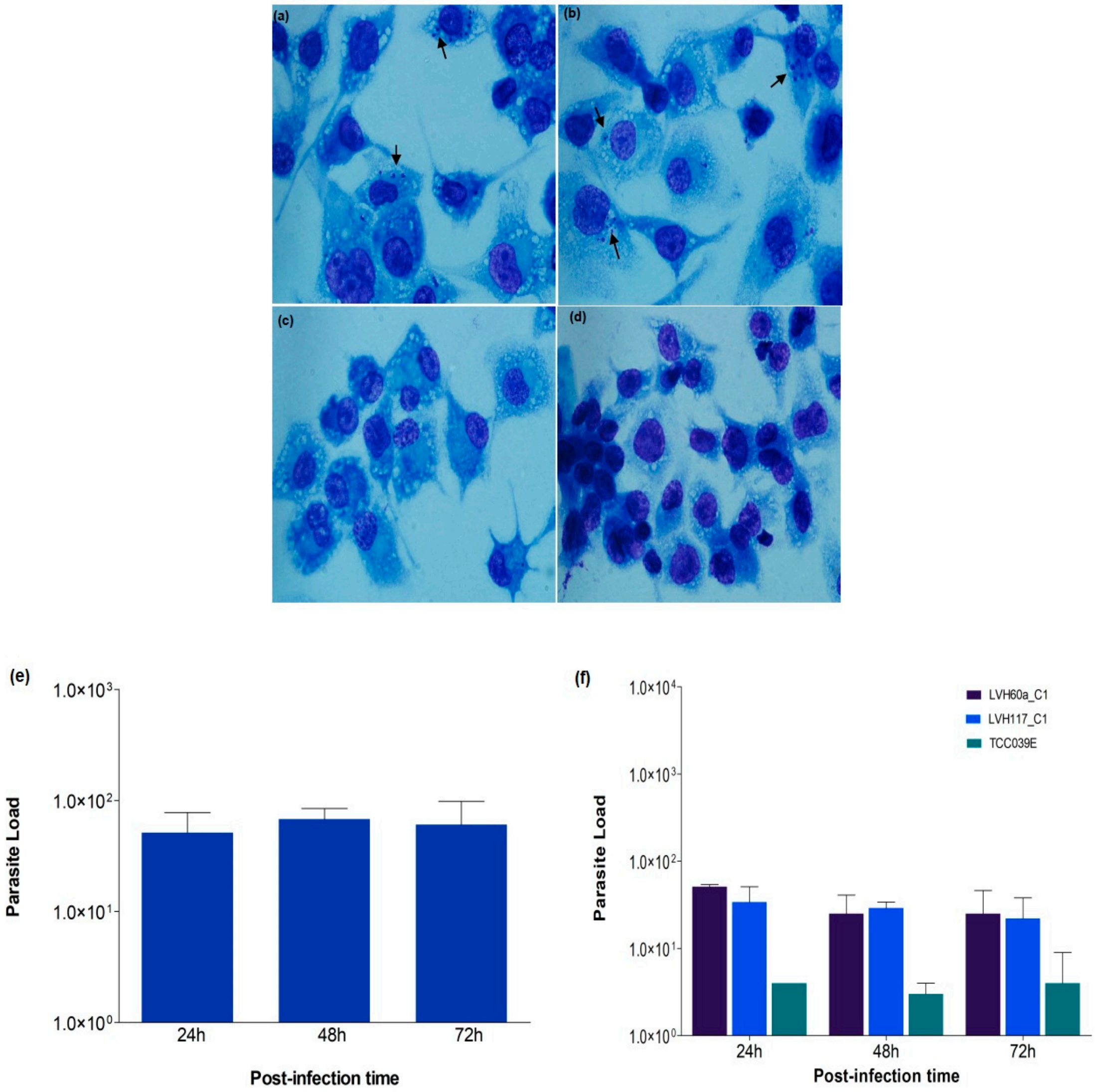

Figure S7: THP-1 macrophages infected with HUUFS14 strain (*L. infantum*), LVH60a_C1, LVH117_C1 (*Crithidia* sp LVH60A) and TCC039E (*C. fasciculata*. (a) *L. Infantum* (HUUFS14 strain), (b) *Crithidia*-like (LVH60a_C1 strain), (c) *C. fasciculata* (TCC039E strain) and (d) control macrophage without any infection in 24 hour post infection respectly. The black arrows indicate the amastigote structures. Light Microscopy Image visualized with oil immersion in a 1000X magnification. (e) Graph of the parasite load measured by microscopy counting the amastigote structures of the HUUFS14 strain in 100 THP-1 macrophages (ratio of amastigotes/ 100 cells). (f)Graph of the parasite load measured by microscopy counting the amastigote structures of the infection with strain LVH60a_C1 (purple color bar), LVH117_C1 (blue color bar) and TCC039E (green color bar) in 100 THP-1 macrophages (ratio of amastigotes/100. The two-away ANOVA test was performed using the Graphpad Prism 5.00 software and no statistically significant difference.

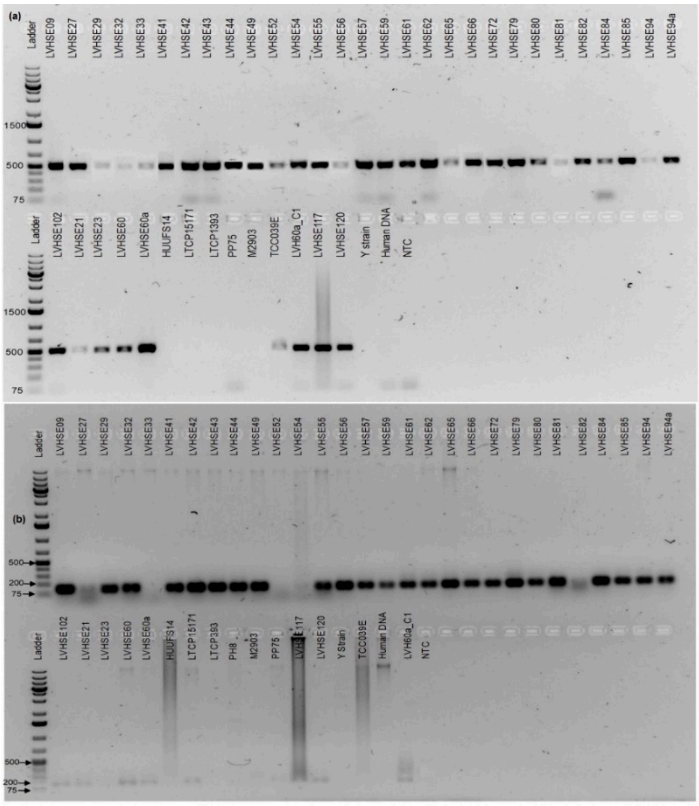

Figure S8: Agarose gel electrophoresis (1%) of amplifications with Crid2.1seq and LVH60a_Tig001 primer. (a) Conventional PCR assay performed with DNA from clinical isolates (~100 ng) with Crid2.1seq primer, specific for *Crithidia* spp. (b) Conventional PCR assay performed with DNA from clinical isolates (~100 ng) with LVH60_Tig0001 primer, specific for *Crithidia*-like. Both assay was used host DNA (human), HUUFS14 and PP75 strain (*L. infantum*), LTCP393, LTCP15171 and M2903 strain (*L. braziliensis*), PH8 (*L. amazonensis*) TCC039E (*C. fasciculata*) samples were also tested. LVH60a_C1 (*Crithidia-like*) sample was used as positive control. NTC: no template control. The image were captured by ChemiDocTM XRS Systems (Bio-Rad) and used software Image Lab. Ladder GeneRuler 1Kb Plus DNA (Thermo Fisher Scientific).

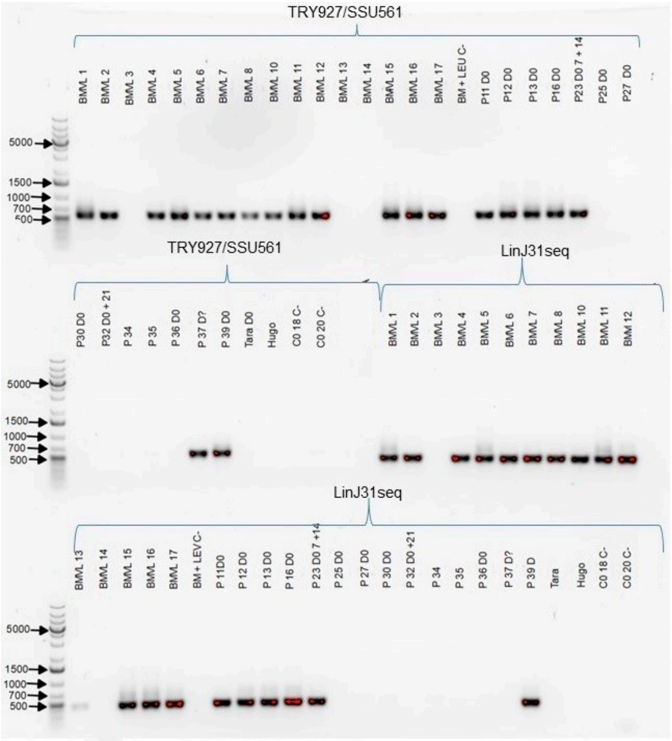

Figure S9: Electrophoresis in agarose gel (1%) of conventional PCR performed with samples of LV patients from HUUFS. Were used TRY927/ SSU561 primer and with the species-specific primer LinJ31seq detecting the LV samples patients bone marrow (BM) and peripheral blood (P). Bone marrow sample not infected by trypanosomatids (BM+LEVC-) used as a negative control. The images were captured by ChemiDocTM XRS Systes (Bio-Rad) and used software Image Lab. Ladder GeneRuler 1Kb Plus DNA (Thermo Fisher Scientific).

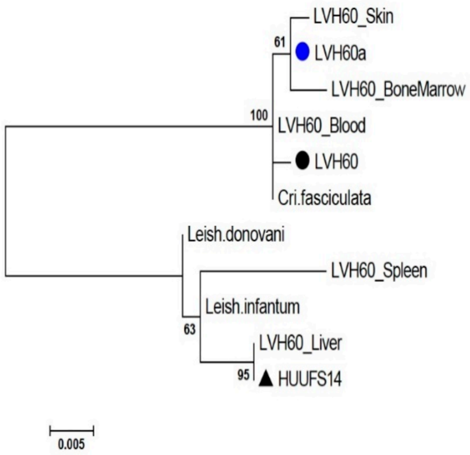

Figure S10: Phylogenetic analyses of small subunit rRNA (ssrRNA) se-quences from LVH60 patient samples. The analysis involved 10 nucleotide sequences and a total of 487 positions in the final dataset; bar at bottom indicates 0.5% nucleotide divergence. Tree was constructed using Maximum Likelihood (ML) method based on the Tamura-Nei model, using *Leishmania* nucleotide sequences retrieved from NCBI according to BLAST-N results. Numbers next to the branches represent the percentage of replicate trees in which the associated taxa clustered together in the bootstrap test (1,000 replicates). All positions with less than 50% site coverage, containing gaps and missing data were eliminated. Black circle indicates parasite strain isolated from patient bone marrow, while blue circle indicates parasite strain isolated from patient

skin lesion. Triangle indicates Brazilian clinical isolate of *L. infantum*. Tissue's patient is indicate after underline in LVH60 denomination. LVH60 case was first reported by Maruyama *et al*. (2019), PMID: 31625841.

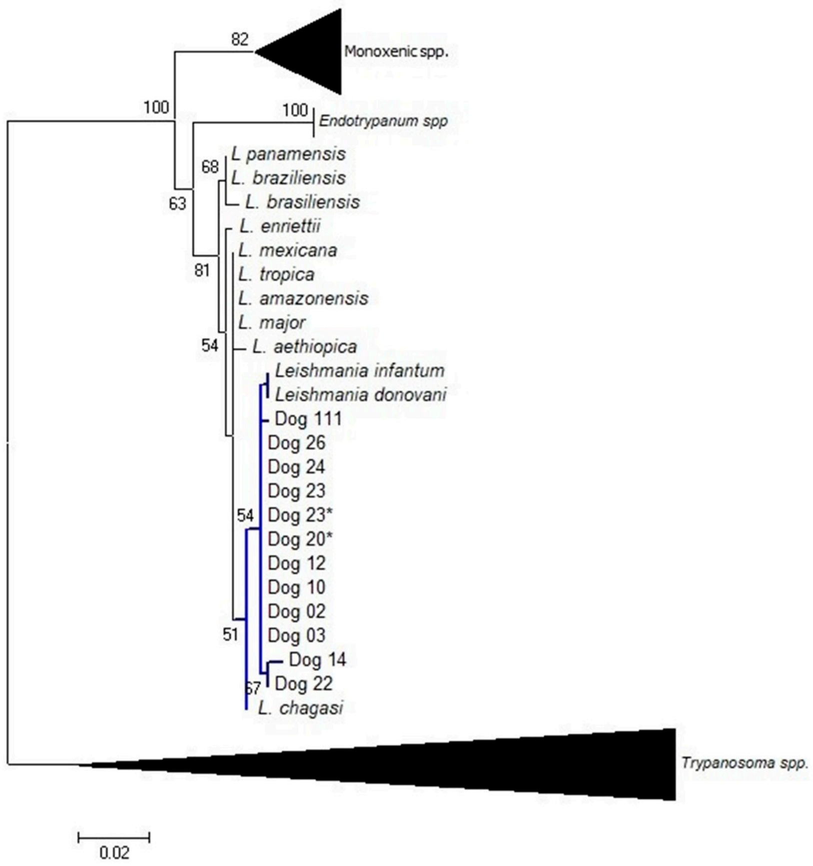

Figure S11: Phylogenetic tree with TRY927/SSU561 rRNA sequences detected in bone marrow and peripheral blood samples of dogs with VL. Phylogenetic tree with TRY/SSU rRNA sequences detected in bone marrow and *peripheral blood samples of dogs with VL, sequences compared with NCBI database (BLASTN) and TriTr.ypDB. Phylogeny generated by the Neighbor-Joining matrix method of pairwise distances estimated by Maximum Composite Likelihood (MCL) Tamura-Nei model with Bootstrap (1000 repliques) by software Mega6. Branches in blue presents the grouping of the samples of dogs in the same branch of the species *L. Infantum* and *L. donovani*.

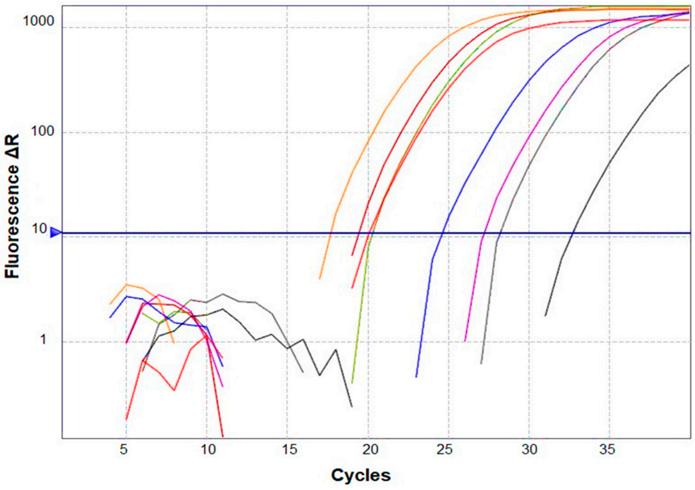

Figure S12: Amplification plot with LinJ31_2420 primer in sand fly samples by qPCR. LinJ31seq primer was used by PCR and the product as a template for reaction by qPCR with LinJ31_2420 primer in sandflies infected with *L. infantum* from the study by Ferreira *et al.* (2018) [27]. Vector 67 orange color amplification; vector 63 reddish brown color amplification, vector 10 olive green color amplification, vector I7 blue color amplification, vector 126 purple color amplification, vector 75 gray color amplification, and vector and vector 119 black color amplification. The HUUFS14 strain (L. infantum) was used as a positive control in the reactions (amplification red color).
