# Supplementary material for: Parasite Detection in Visceral Leishmaniasis Samples by Dye-Based qPCR Using New Gene Targets of Leishmania infantum and Crithidia
Source: Trop Med Infect Dis. 2023 Aug 8;8(8):405. doi: 10.3390/tropicalmed8080405 (PMC10457869; doi:10.3390/tropicalmed8080405)
Supplement: Supplementary file 1 [file tropicalmed-08-00405-s001.zip › tropicalmed-2509720 - Supplemental-Tables_Takamyia-et-al-NTT-SRM_v10.pdf]

Supplementary Tables

Table S1: Calculations of DNA mass equivalent to one parasite.

Parasite mass (m) = [n x 1.096e-21 g/bp#] x ploidy + 15.8% kDNA mass

Specie	Calculations
L. infantum	Parasite mass of one *L. infantum*:<br>m= 32.13e6 bp x 1.096e-21 g/ bp x 2 (n)= 70.4 fg<br><br>70.4 + 15.8% kDNA= 81.52 e-15g
Crithidia sp LVH-60A	Parasite mass of one *Crithidia sp* LVH-60A<br>(LVH60a)<br><br>m= 34.4e6 bp x 1.096e-21 g/ bp x 2 (n)= 75.4 fg<br><br>75.4 fg + 15.8%*= 87.3 e-15g

*Percentage of *Crithidia sp* LVH-60A kDNA mass was considered to be equal to *Leishmania* spp.

Table S1: Calculation of parasite mass of a C. *fasciculata* based on genome size in https://www.ncbi.nlm.nih.gov/datasets/taxonomy/5656/

Table S2: Serial dilution for qPCR standard curve according to the calculation of the DNA mass equivalent to one parasite of *L. infantum*.

# Parasites		Required mass ofparasite DNA (g)		Final concentration of the parasite (g/uL)
10^6		81.5 e-09		40.7 e-09
10^5		81.5 e-10		40.7 e-10
10^4	x 81.52 e-15g	81.5 e-11	/ 2μL	40.7 e-11
10^3		81.5 e-12		40.7 e-12
10^2		81.5 e-13		40.7 e-13
10^1		81.5 e-14		40.7 e-14
10^0		81.5 e-15		40.7 e-15

Dilution	Parasite DNA for dilution	[ ] initial g/uL C1	DNA volume V1	Dilution volume	Final volume V2	[ ] final g/uL C2	Estimate of the number of parasites in 2 uL
1	Stock					40.7 e-09	10^6
2	Tube 1	40.7 e-09	10	90	100	40.7 e-10	10^5
3	Tube 2	40.7 e-10	10	90	100	40.7 e-11	10^4
4	Tube 3	40.7 e-11	10	90	100	40.7 e-12	10^3
5	Tube 4	40.7 e-12	10	90	100	40.7 e-13	10^2
6	Tube 5	40.7 e-13	10	90	100	40.7 e-14	10^1
7	Tube 6	40.7 e-14	10	90	100	40.7 e-15	10^0

Serial dilution according to the calculation of the DNA mass equivalent to one parasite of *Crithidia sp* LVH-60A.

**# Parasites**		**Required mass of parasite DNA (g)**		**Final concentration of the parasite (g/uL)**
10^6		87.3 e-09		43.6 e-09
10^5		87.3 e-10		43.6 e-10
10^4	x 87.3 e-15g	87.3 e-11	/ 2µL	43.6 e-11
10^3		87.3 e-12		43.6 e-12
10^2		87.3 e-13		43.6 e-13
10^1		87.3 e-14		43.6 e-14
10^0		87.3 e-15		43.6 e-15

**Dilution**	**Parasite DNA for dilution**	**[ ] initial g/uL C1**	**DNA volume V1**	**Dilution volume**	**Final volume V2**	**[ ] final g/uL C2**	**Estimate of the number of parasites in 2 uL**
1	Stock					43.6 e-09	10^6
2	Tube 1	43.6 e-09	10	90	100	43.6 e-10	10^5
3	Tube 2	43.6 e-10	10	90	100	43.6 e-11	10^4
4	Tube 3	43.6 e-11	10	90	100	43.6 e-12	10^3
5	Tube 4	43.6 e-12	10	90	100	43.6 e-13	10^2
6	Tube 5	43.6 e-13	10	90	100	43.6 e-14	10^1
7	Tube 6	43.6 e-14	10	90	100	43.6 e-15	10^0

Table S3: Table of proportions of the number of parasites for the Spike-in assay (used in Figure6):

**Number of parasites (*L. infantum*) + background host [4 ng/ μL]**	**Number of parasites (*Crithidia sp* LVH-60A) + background host [4 ng/ μL]**
5×10^2	0
5×10^4	0
5×10^2	5×10^2
5×10^2	5×10^4
0	5×10^2
0	5×10^4
5×10^2	5×10^2
5×10^4	5×10^2

OBS: Final volume of each reaction 10μL.

Table S4: Informations of gene ID, chromosome, location genome, number copies of p-nitrophenylphosphatase putative gene in *Leishmania* spp.

Species	Gene code	Gene description	Chr.	Location	Number copies gene
L. infantum (JPCM5)	LinJ31_2420	p-nitrophenylphosphatase - putative	31	start: 1 200 125, end: 1 201 141, on reversed strand of LinJ.31	1
L. donovani (BPK282A1)	LdBPK_312410.1	p-nitrophenylphosphatase - putative	31	start: 1 165 515, end: 1 166 756, on reversed strand of Ld31_v01s1	1
L. braziliensis (MHOM/BR/75/ M2904)	LbrM.31.2620	p-nitrophenylphosphatase - putative	31	start: 1 201 306, end: 1 202 400, on reversed strand of LbrM.31/ start: 1 205 959, end: 1 206 693, on reversed strand of LbrM.31	2
L. amazonensis (MHOM/BR/719 73/M2269)	LAMA_000645300	Haloacid dehalogenase-like hydrolase/ HAD-hyrolase-like/ Mitochondrial PGP phosphatase, putative	Not Assigned	start: 1382, end: 2476, on reversed strand of KE390335.1	1
L. mexicana (MHOM/GT/200 1/U1103)	LmxM.30.2340	p-nitrophenylphosphatase - putative	30	start: 1 118 250, end: 1 119 344, on reversed strand of LmxM.30	1
L. major (Friedlin)	LmjF.31.2340	p-nitrophenylphosphatase - putative	31	start: 1 152 129, end: 1 163 738, on reversed strand of LmjF.31	1

Chr.: Chromosome.

Table S5: Informations of gene ID, chromosome, location genome, number copies of Catalase gene in *Crithidia sp* LVH-60A, *C. fasciculata* and *Leptomonas* spp.

Species	Gene code	Gene description	Chr.	Location
Crithidia fasciculata (CfCl)	CFAC1_250006200#	Catalase/Catalase-related immune-responsive, putative	25	start: 20 383, end: 21 864, on reversed strand of CfaC1_25
	CFAC1_280006600#	Catalase/Catalase-related immune-responsive, putative	28	start: 26 846, end: 28 327, on reversed strand of CfaC1_28
	CFAC1_290005500#	Catalase/Catalase-related immune-responsive, putative	29	start: 16 681, end: 18 162, on reversed strand of CfaC1_29
	CFAC1_160031400#	Catalase/Catalase-related immune-responsive, putative	16	start: 799 545 , end: 801 026, on forward strand of CfaC1_16
Crithidia sp LVH-60A (LVH60a_C1)	CP119667.1	Catalase - putative	10	start: 40 0307, end: 400394
	CP119668.1	Catalase - putative	36	start: 27609. end: 27522
	CP119663.1	Catalase - putative	5	start: 271437, end: 271524
	CP119644.1	Catalase - putative	32	start: 1430866, end:
Leptomonas pyrrhocoris (H10)	LpyrH10_15_0020#	Catalase	15	start: 3623, end: 5209, on reversed strand of LpyrH10_15
Leptomonas seymouri (ATCC_30220)	Lsey_0026_0490#	Catalase	Not Assigned	start: 135 714 , end: 137 201, on forward strand of Lsey_0026

Chr.: Chromosome ;#TriTryDB reference

Table S6: Coefficient values for qPCR standard curves using hosts DNA as background in reactions. Assays were performed in two qPCR instruments of different brands with curves set up six points in 10-fold serial dilutions.

Primer LinJ31_2420	Source of DNA background	Slope	R^{2*}	Eff%**
7500 Fast Dx Real Time PCR Machine (Thermo Fisher Scientific)	DNA Human	-3.77	0.949	111.65
	DNA Dog	-3.53	0.945	113.85
	DNA Cat	-3.12	0.988	108.80
	DNA J774	-3.82	0.952	114.88
AriaMx Real- Time PCR System (Agilent)	DNA Human	-3.22	0.997	104.26
	DNA Dog	-3.35	0.971	114.50
	DNA Cat	-3.35	0.998	98.46
	DNA J774	-3.29	0.999	101.29

*coefficient of determination; ** percentage of reaction efficiency

Primer Catalase_LVH60-12060_1F	Spike-in Assay	Slope	R^2	Eff%
7500 Fast Dx Real Time PCR Machine (Thermo Fisher Scientific)	DNA Human	-3.16	0.953	107.13
	DNA Dog	-3.02	0.945	113.85
	DNA Cat	-3.47	0.981	94.10
	DNA J774	-3.01	0.952	114.88
AriaMx Real- Time PCR System (Agilent)	DNA Human	-3.28	0.993	101.62
	DNA Dog	-3.05	0.978	112.88
	DNA Cat	-3.51	0.977	119.23
	DNA J774	-3.30	0.998	100.76

*coefficient of determination; ** percentage of reaction efficiency

Table S7: PCR results with species-specific primers and molecular typing by Sanger sequencing.

Samples	Tissue	SSU rRNA (18S) marker	PCR Linj31Seq	PCR Crid2.1Seq	Clinical isolate (ID)
BM.VL1	Bone Marrow	*L. infantum*	P	NA	LVHSE09
BM.VL2	Bone Marrow	*L. infantum*	P	NA	LVHSE27
BM.VL4	Bone Marrow	*L. infantum*	P	NA	LVHSE32
BM.VL5	Bone Marrow	*L. infantum*	P	NA	LVHSE43
BM.VL6	Bone Marrow	*L. infantum*	P	NA	LVHSE52
BM.VL7	Bone Marrow	*L. infantum*	P	NA	LVHSE54
BM.VL8	Bone Marrow	*L. infantum*	P	NA	No
BM.VL10	Bone Marrow	*L. infantum*	P	NA	LVHSE59
BM.VL11	Bone Marrow	*L. infantum*	P	NA	LVHSE61
BM.VL12	Bone Marrow	*L. infantum*	P	NA	LVHSE62
BM.VL15	Bone Marrow	*L. infantum*	P	NA	LVHSE49
BM.VL16	Bone Marrow	*L. infantum*	P	NA	LVHSE50
BM.VL17	Bone Marrow	#	P	NA	No
BMVL 60*	Bone Marrow	*Crithidia* sp. LVH-60A	N	NA	LVHSE60
GSJ	Bone Marrow	*Crithidia* sp. LVH-60A	NP	NA	LVHSE148
JFJ	Bone Marrow	*L. infantum*	NP	NA	No
LBV	Bone Marrow	*L. infantum*	NP	NA	No
AV	Bone Marrow	*L. infantum*	NP	NA	No
P13D0*	Peripheral blood	*L. infantum*	P	NA	LVHSE60
P11D0	Peripheral blood	*L. infantum*	P	NA	No
P12D0	Peripheral blood	*L. infantum*	P	NA	No
P16D0	Peripheral blood	*L. infantum*	P	NA	LVHSE101
P23D0+7+14	Peripheral blood	*L. infantum*	P	NA	No
P37D	Peripheral blood	#	N	NA	No
P39D	Peripheral blood	*L. infantum*	P	NA	No

*Relapsed patient (study by Maruyama *et al*. 2019); #: TRY927/SSU561 poor quality sequencing; P: PCR positive; N: PCR negative; NP: Not performed; NA: not compatible with amplicon band size; ID: isolate identification LVHSE

Table S8: Table of PCR results with LinJ31seq and Crid2.1seq primers. TRY/SSU column displays molecular typing by amplicon analysis (sequencing Sanger) of clinical isolates.

Clinical isolates	Origin of the isolate	PCR Linj31Seq	PCR Crid2.1Seq	TRY/SSU Amplicons
LVHSE09[a]	Bone marrow	N	P	*Crithidia sp*
LVHSE21	Bone marrow	N	P	*Crithidia sp*
LVHSE23[a]	Bone marrow	N	P	*Crithidia sp*
LVHSE27	Bone marrow	N	P	*Crithidia sp*
LVHSE29	Bone marrow	N	P	*Crithidia sp*
LVHSE32	Bone marrow	N	P	*Crithidia sp*
LVHSE33	Bone marrow	N	P	*Crithidia sp*
LVHSE41	Bone marrow	N	P	*Crithidia sp*
LVHSE43[a]	Bone marrow	N	P[#]	*Crithidia sp*
LVHSE44	Bone marrow	N	P	*Crithidia sp*
LVHSE49	Bone marrow	N	P	*Crithidia sp*
LVHSE52	Bone marrow	N	P	*Crithidia sp*
LVHSE54	Bone marrow	N	P	*Crithidia sp*
LVHSE55	Bone marrow	N	P	*Crithidia sp*
LVHSE56	Bone marrow	N	P	*Crithidia sp*
LVHSE57	Bone marrow	P	P[#]	*Crithidia sp*
LVHSE59	Bone marrow	P	P	*Crithidia sp*
LVHSE61	Bone marrow	N	P	*Crithidia sp*
LVHSE62	Bone marrow	N	P	*Crithidia sp*
LVHSE65	Bone marrow	N	P	*Crithidia sp*
LVHSE66	Bone marrow	P	P	*Crithidia sp*
LVHSE72	Bone marrow	N	P	*Crithidia sp*
LVHSE79	Bone marrow	N	P	*Crithidia sp*
LVHSE80	Bone marrow	N	P	*Crithidia sp*
LVHSE81	Bone marrow	N	P	*Crithidia sp*
LVHSE82	Bone marrow	N	P	*Crithidia sp*
LVHSE84	Bone marrow	N	P	*Crithidia sp*
LVHSE85	Bone marrow	N	P	*Crithidia sp*
LVHSE94	Bone marrow	N	P[#]	*Crithidia sp*
LVHSE94a	Skin tissue	N	P	*Crithidia sp*
LVHSE101[a]	Bone marrow	N	P	*Crithidia sp*
LVHSE103	Bone marrow	N	P	*Crithidia sp*
LVHSE105	Bone marrow	N	P	*Crithidia sp*
LVHSE107	Bone marrow	N	P	*
LVHSE109	Bone marrow	N	P	*Crithidia sp*
LVHSE110	Bone marrow	N	P	*Crithidia sp*
LVHSE115	Bone marrow	P	N	*
LVHSE117[b]	Bone marrow	P	P	*Crithidia sp*
LVHSE119	Bone marrow	P	N	*L. infantum*
LVHSE120[b]	Spleen	N	P	*Crithidia sp*
LVHSE132	Bone marrow	P	N	*
LVHSE133	Bone marrow	P	N	*L. infantum*
LVHSE134	Bone marrow	N	P	*Crithidia sp*
LVHSE135	Bone marrow	N	P	*Crithidia sp*
LVHSE137	Bone marrow	N	P	*
LVHSE138	Bone marrow	N	P	*
LVHSE139	Bone marrow	N	P	*Crithidia sp*
LVHSE140	Bone marrow	N	P	*Crithidia sp*
LVHSE141	Bone marrow	N	P	*Crithidia sp*
LVHSE142	Bone marrow	N	P	*
LVHSE146	Bone marrow	N	P	*
LVHSE148	Bone marrow	N	P	*
LVHSE149	Bone marrow	N	P	*Crithidia sp*
LVHSE153	Bone marrow	P[#]	N	*

LVHSE159	Bone marrow	P#	N	*
LVHSE160	Bone marrow	P#	N	*L. infantum*
LVHSE161[b#]	Bone marrow	P	N	*L. infantum*
LVHSE162	Bone marrow	P	N	*
LVHSE161a_N1[b#]	Skin nodule	P	N	*L. infantum*
LVHSE161a_N2[b#]	Skin nodule	P	N	*L. infantum*
LVHSE60**	Bone marrow	N	P	*Crithidia sp*
LVHSE60a**	Skin papule	N	P	*Crithidia sp*

*Poor quality sequencing. [a]isolate from relapsed patient. [b]Isolate from same patient in year 2017(study by Rogerio *et al*. 2023) (accession numbers OQ581230.1 and OQ581233.1); [b#]Relapsed patient in year 2020 (same patient of the clinical isolate LVHSE117, study by Rogerio *et al*. 2023) (accession numbers OQ581236.1 and OQ581240.1). **Relapsed patient (study by Maruyama *et al*. 2019) (Number accession OQ581229.1 and OQ581228.1.

Table S9. Accession numbers of small subunit rRNA (ssrRNA) sequences used in phylogenetic analysis displayedin Supplementary Figure S8 and Figure S9.

**Sequences retrieved from NCBI**	
Crithidia fasciculata	Y00055.1
Leishmania infantum	XR_001203206.1
Leishmania chagasi	KU948455.1
Leishmania donovani	FR799614.1
**Sequences generated in this work**	
Trypanosomatidae sequences from human samples (Figure S8).	http://www.ebi.ac.uk/ena/data/view/PRJEB25906
Trypanosomatidae sequences from dog samples (Figure S9).	To be released soon
